# Supplementary material for: Isolable f-element diphosphene complexes by phosphinidene group transfer and coupling at uranium
Source: Nat Commun. 2025 Nov 25;16:10445. doi: 10.1038/s41467-025-65433-4 (PMC12647618; doi:10.1038/s41467-025-65433-4)
Supplement: Supplementary file 1 — Supplementary Information [file 41467_2025_65433_MOESM1_ESM.pdf]

– Supplementary Information –

Isolable f-Element Diphosphene Complexes by Phosphinidene Group Transfer and Coupling at Uranium

Jingzhen Du,[1,2] Thayalan Rajeshkumar,[3] John A. Seed,[1] Ashley J. Wooles,[1] Laurent Maron,[3]* and Stephen T. Liddle[1]*

[1] Department of Chemistry and Centre for Radiochemistry Research, The University of Manchester, Oxford Road, Manchester, M13 9PL, UK.

[2] Present Address: College of Chemistry, Zhengzhou University, Zhengzhou, 450001, China.

[3] LPCNO, CNRS & INSA, Université Paul Sabatier 135 Avenue de Rangueil, 31077 Toulouse, France.

*e-mail: laurent.maron@irsamc.ups-tlse.fr and steve.liddle@manchester.ac.uk

SUPPLEMENTARY CRYSTALLOGRAPHIC ALERTS

Complex 6

PLAT972_ALERT_2_B Check Calc'd Resid. Dens. 0.93 Å From U1 -2.61 e$Å^{-3}$

PLAT972_ALERT_2_B Check Calc'd Resid. Dens. 0.72 Å From U1 -2.55 e$Å^{-3}$

Author Response: Residual densities are close to a heavy atom, and are present despite applying absorption corrections. They are not chemically significant.

Complex 7

PLAT915_ALERT_3_B No Flack x Check Done: Low Friedel Pair Coverage 44%

Author Response: The absolute configuration of the molecules was of no interest so Friedel Pairs were treated as equivalent during the data collection.

Complex 8

PLAT971_ALERT_2_A Check Calc'd Resid. Dens. 0.76 Å From U1 4.31 e$Å^{-3}$

PLAT971_ALERT_2_A Check Calc'd Resid. Dens. 0.86 Å From U1 4.28 e$Å^{-3}$

PLAT971_ALERT_2_B Check Calc'd Resid. Dens. 1.11 Å From P1 3.10 e$Å^{-3}$

PLAT971_ALERT_2_B Check Calc'd Resid. Dens. 0.76 Å From U1 2.93 e$Å^{-3}$

PLAT971_ALERT_2_B Check Calc'd Resid. Dens. 0.82 Å From U1 2.88 e$Å^{-3}$

Author Response: Residual densities are close to a heavy atoms, and are present despite applying absorption corrections. They are not chemically significant.

SUPPLEMENTARY FIGURES

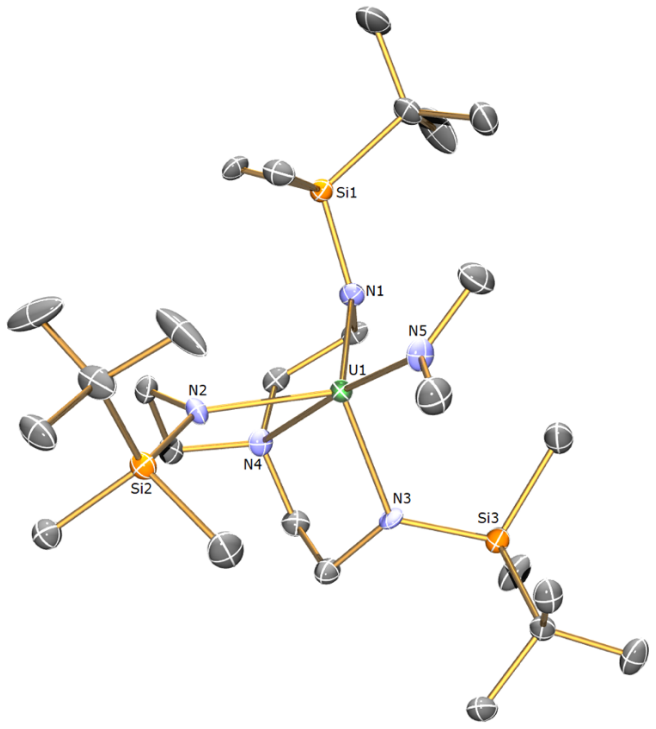

Supplementary Figure 1. Molecular structure of **4** at 150 K with selected atom labels with displacement ellipsoids set at 40% and hydrogen atoms omitted for clarity. CCDC2424900.

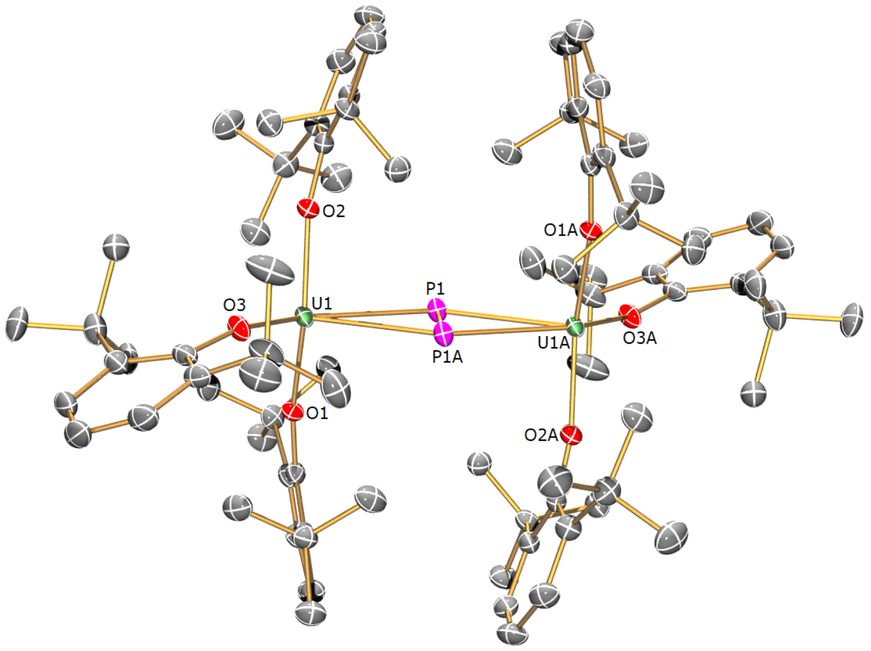

Supplementary Figure 2. Molecular structure of **6** at 150 K with selected atom labels with displacement ellipsoids set at 40%. Disordered components, lattice solvent and hydrogen atoms are omitted for clarity. CCDC2424901.

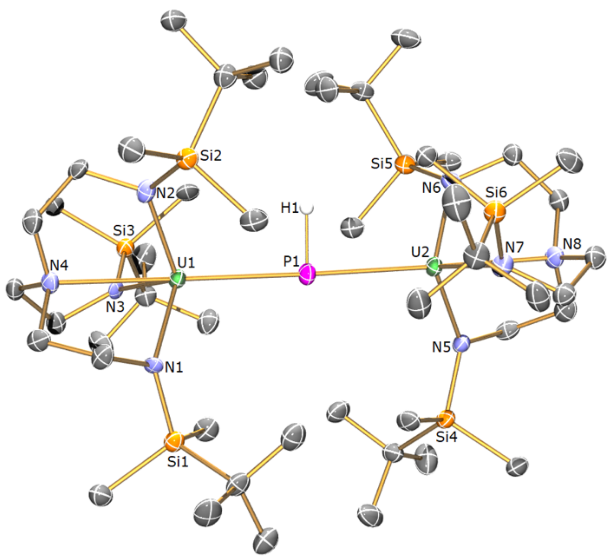

Supplementary Figure 3. Molecular structure of **7** at 150 K with selected atom labels with displacement ellipsoids set at 40%. Hydrogen atoms except for H1 are omitted for clarity. There are two independent molecules in the asymmetric unit, as they are very similar to each other only one molecule is shown here. CCDC2424902.

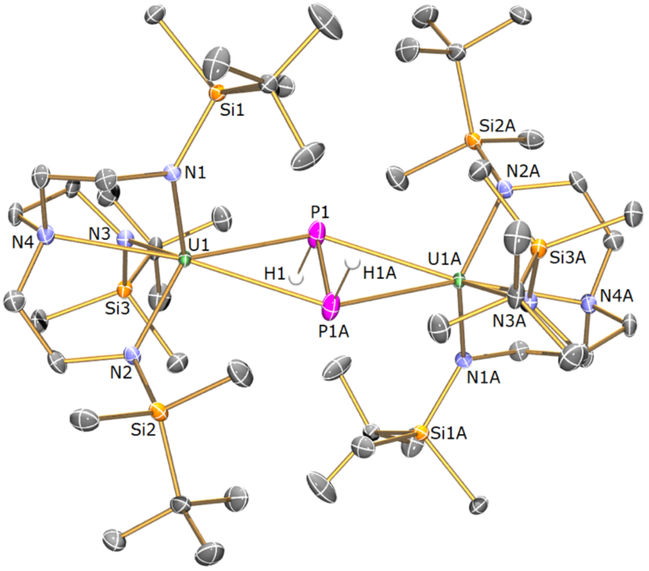

Supplementary Figure 4. Molecular structure of **8** at 100 K with selected atom labels with displacement ellipsoids set at 40%. Hydrogen atoms except for H1 and H1A and lattice solvent are omitted for clarity. CCDC2424903.

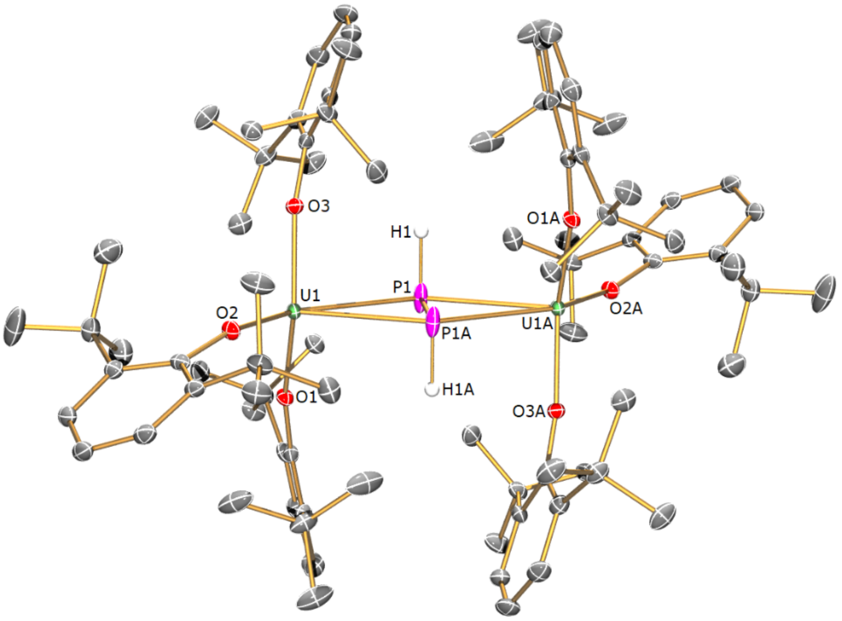

Supplementary Figure 5. Molecular structure of **9** at 100 K with selected atom labels with displacement ellipsoids set at 40%. Hydrogen atoms except for H1 and H1A and lattice solvent are omitted for clarity. CCDC2424904.

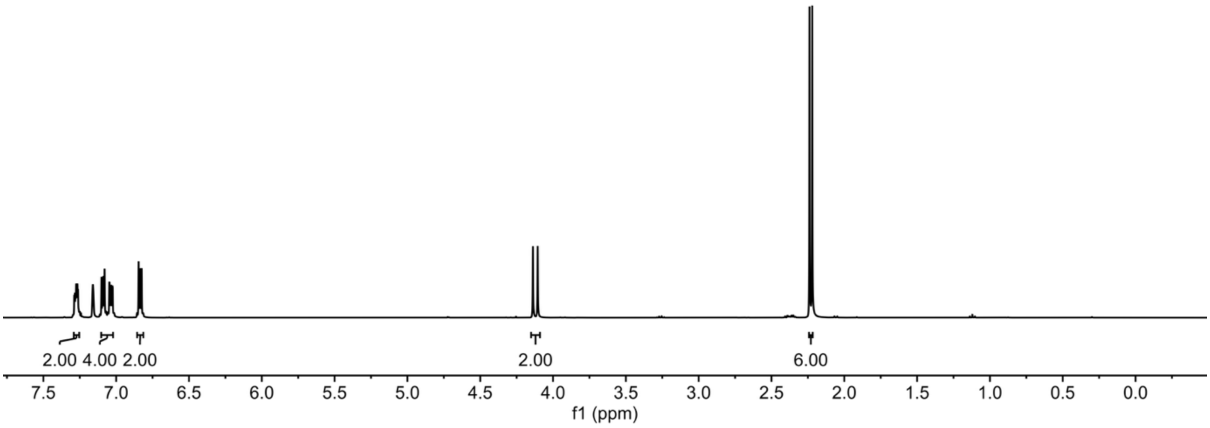

Supplementary Figure 6. ^{1}H NMR (400 MHz, C_6D_6, 298 K) spectrum of **1**.

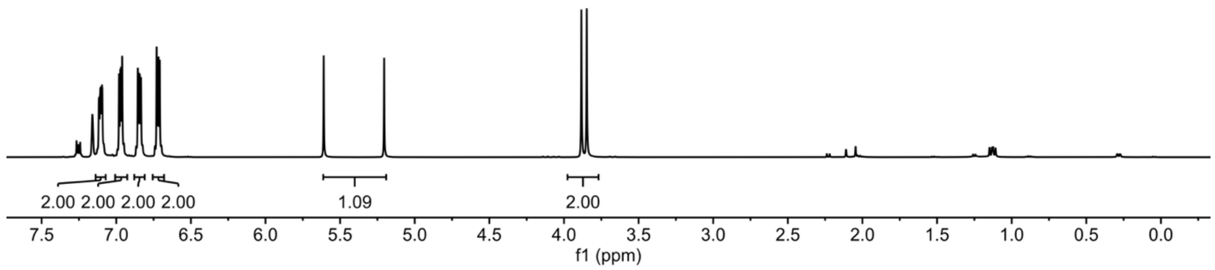

Supplementary Figure 7. ^{1}H NMR (400 MHz, C_6D_6, 298 K) spectrum of **2**.

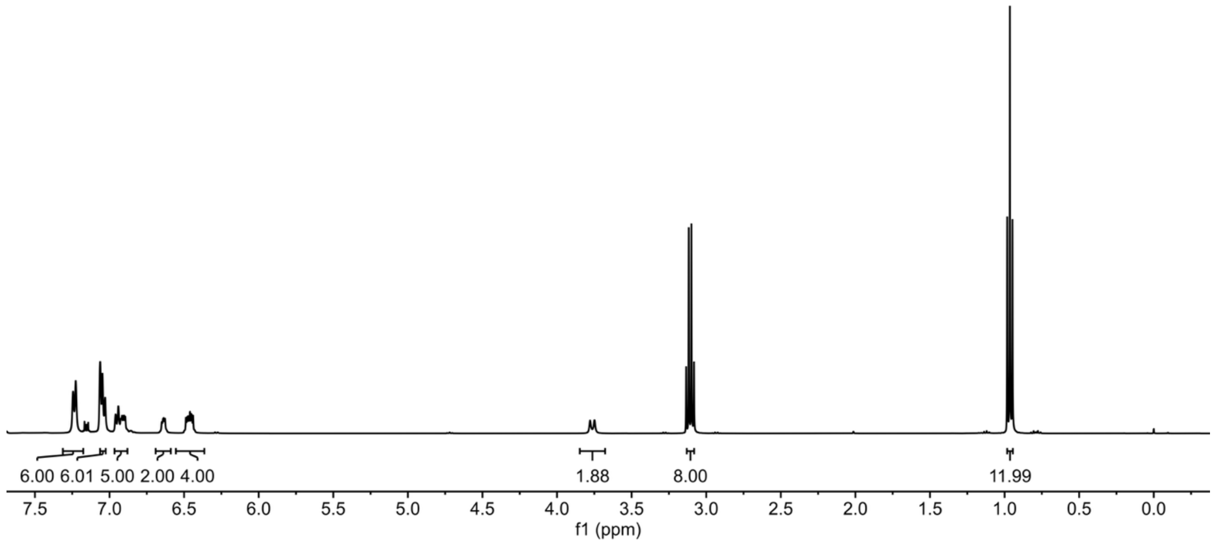

Supplementary Figure 8. ^{1}H NMR (400 MHz, C_6D_6, 298 K) spectrum of PDBN-(BPh_3)Na(OEt_2)$_2$.

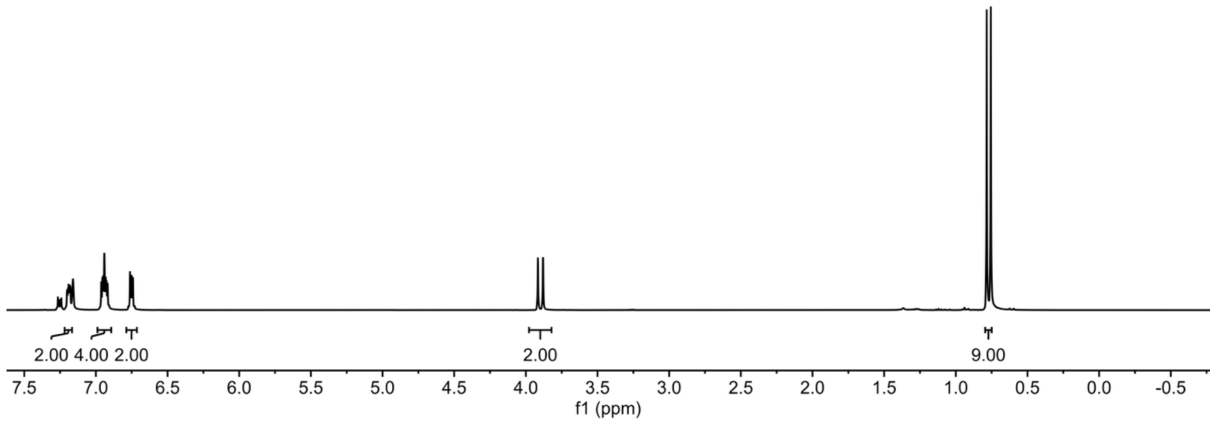

Supplementary Figure 9. ^{1}H NMR (400 MHz, C_6D_6, 298 K) spectrum of PDBN-Bu^t.

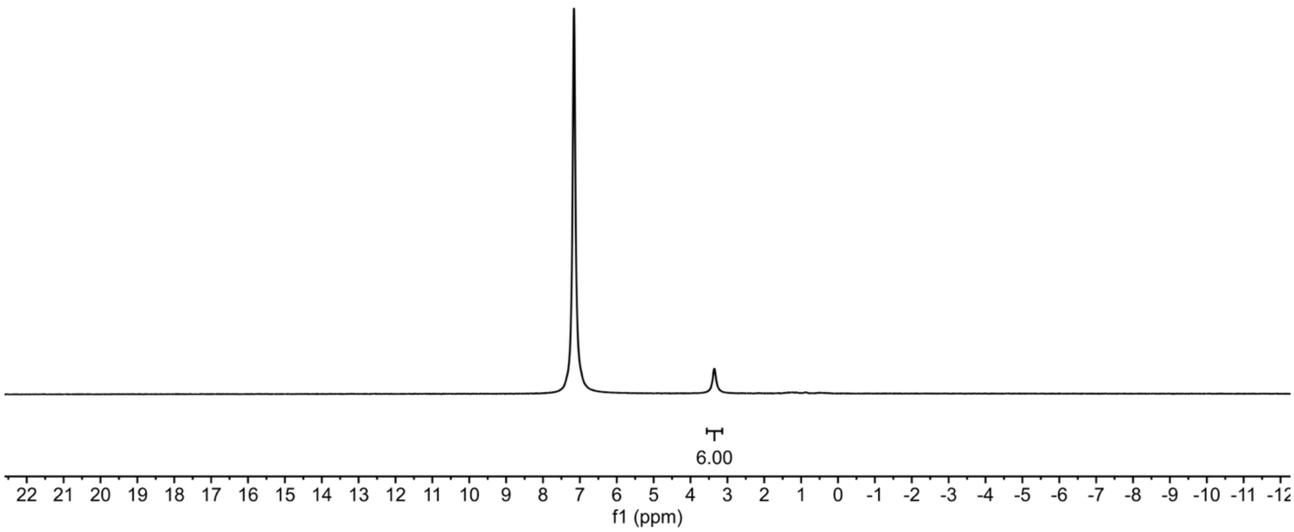

Supplementary Figure 10. ^{1}H NMR (400 MHz, C_6D_6, 298 K) spectrum of $UI_3(1,4\text{-dioxane})_{1.5}$.

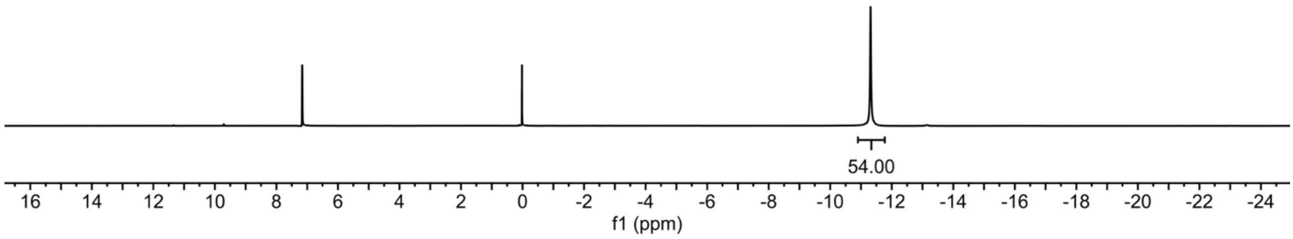

Supplementary Figure 11. ^1H NMR (400 MHz, C_6D_6, 298 K) spectrum of $[U\{N(SiMe_3)_2\}_3]$.

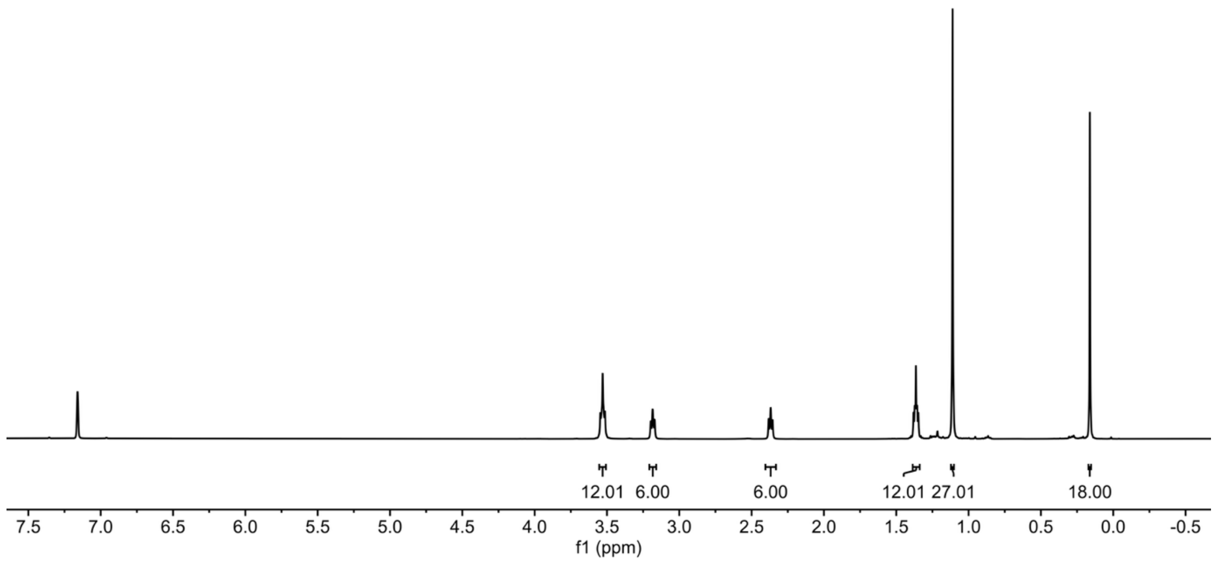

Supplementary Figure 12. ^1H NMR (400 MHz, C_6D_6, 298 K) spectrum of $Tren^{DMBS}Li_3(THF)_3$.

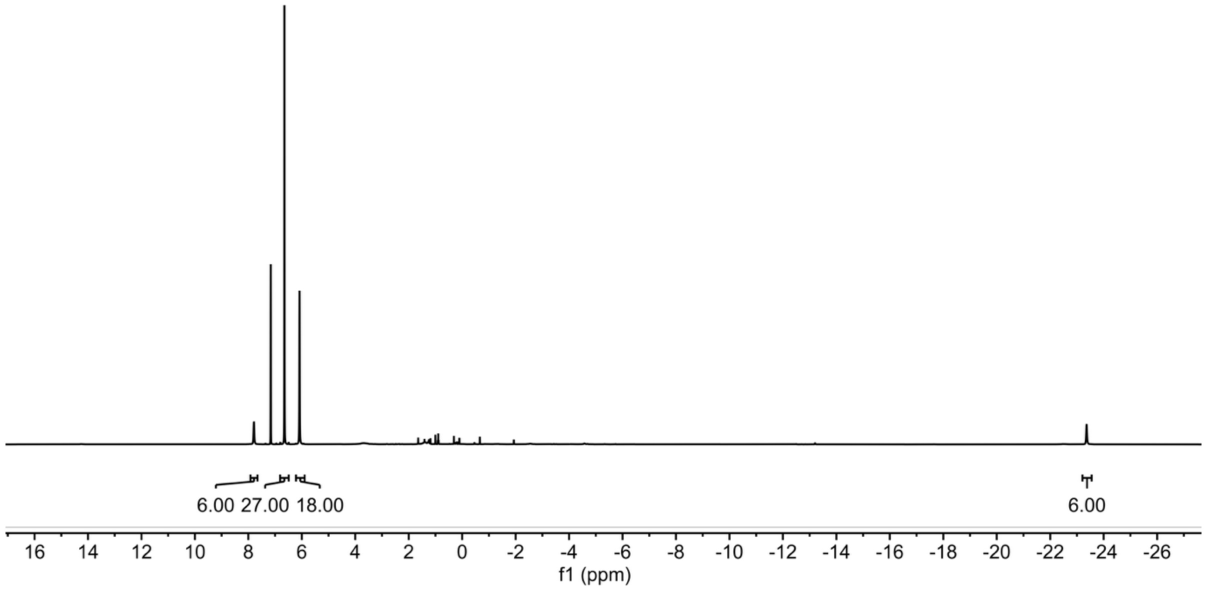

Supplementary Figure 13. ^{1}H NMR (400 MHz, C_6D_6, 298 K) spectrum of [(Tren^{DMBS})U^{IV}(Cl)].

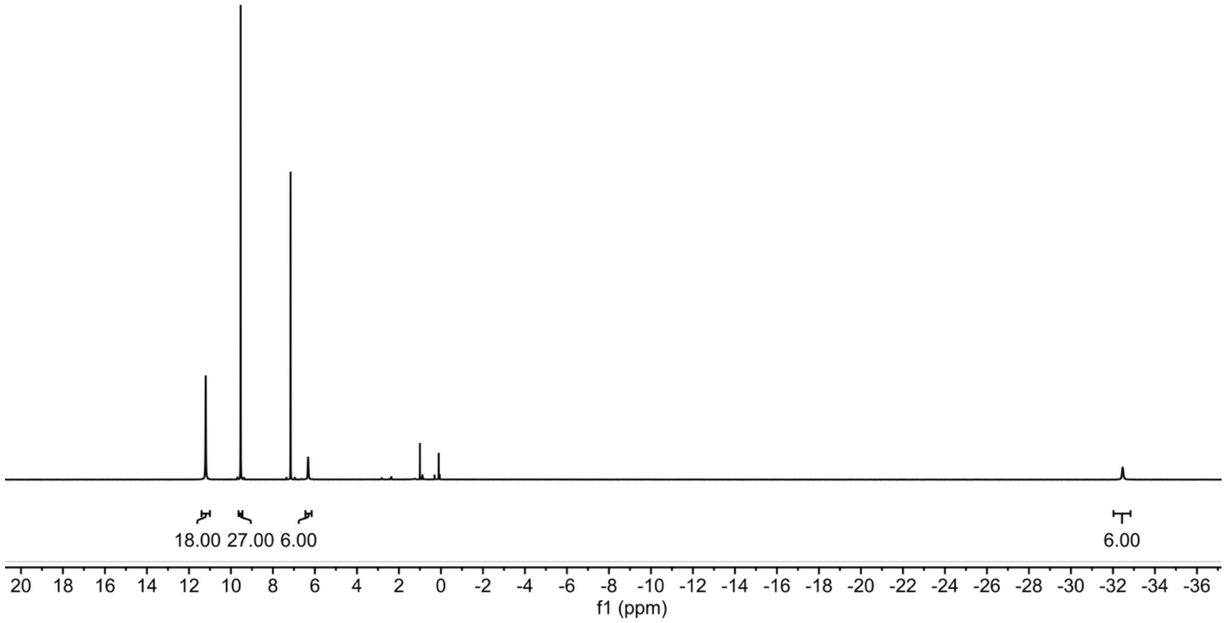

Supplementary Figure 14. ^{1}H NMR (400 MHz, C_6D_6, 298 K) spectrum of [(Tren^{DMBS})U^{IV}(I)].

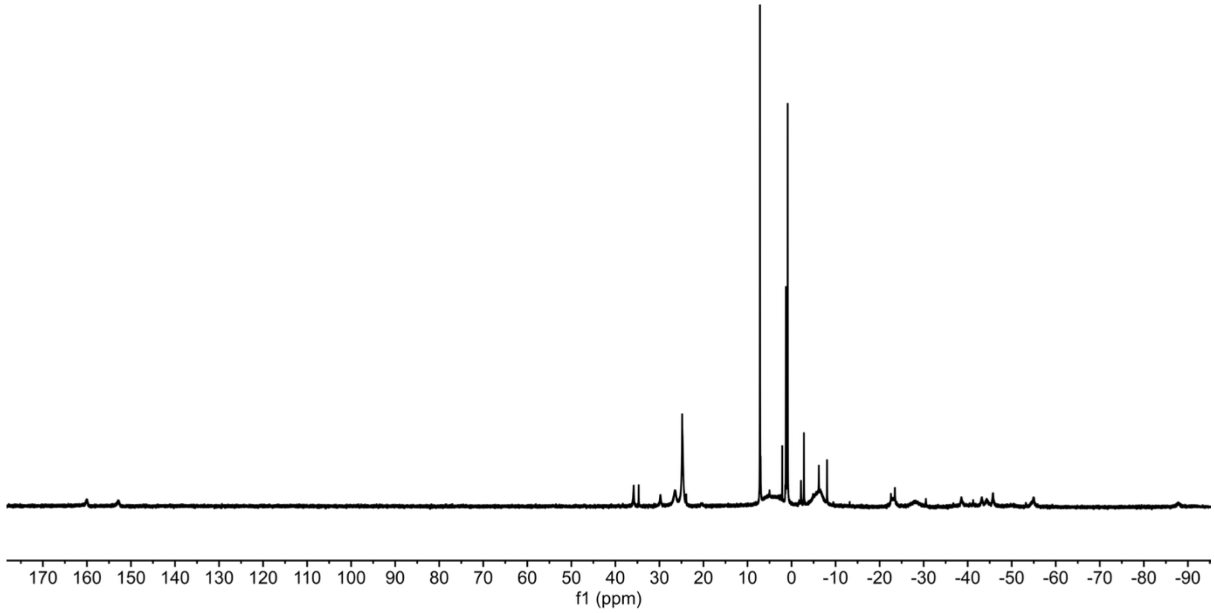

Supplementary Figure 15. ^{1}H NMR (400 MHz, C_6D_6, 298 K) spectrum of [{[K(Toluene)] [(Tren^{DMBS})U^{Cyclomet}]}$_2$].

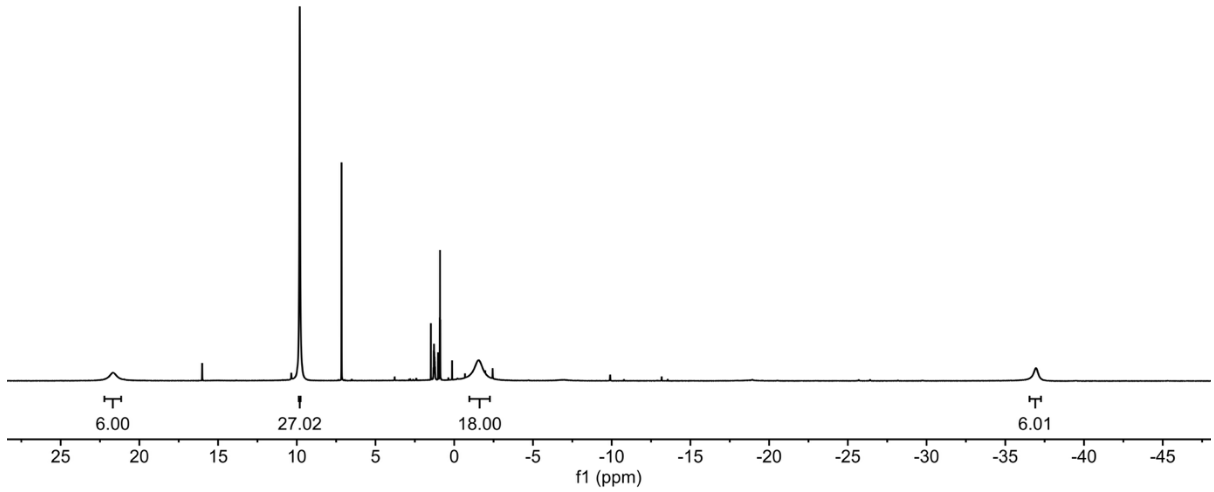

Supplementary Figure 16. ^{1}H NMR (400 MHz, C_6D_6, 298 K) spectrum of **3**.

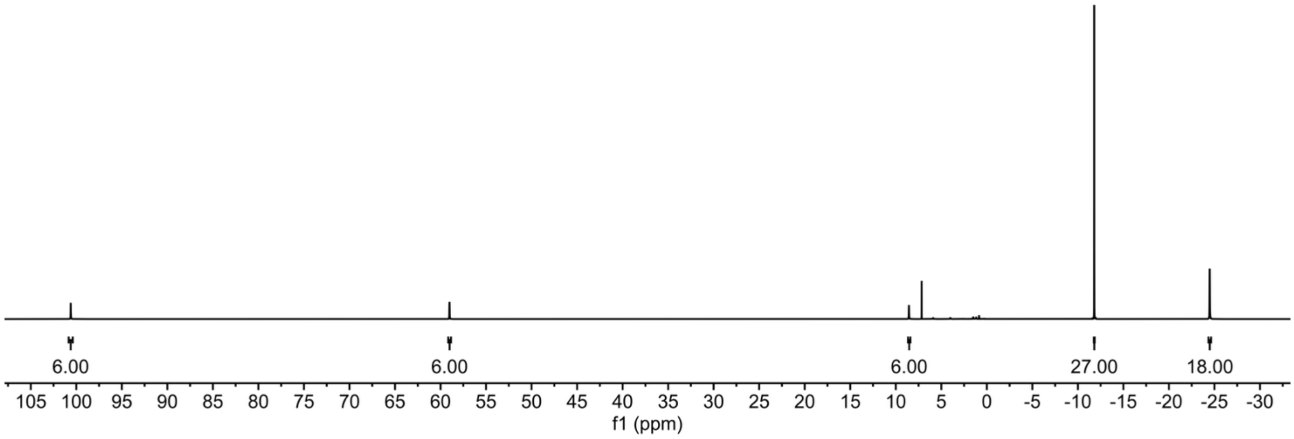

Supplementary Figure 17. ^{1}H NMR (400 MHz, C_6D_6, 298 K) spectrum of **4**.

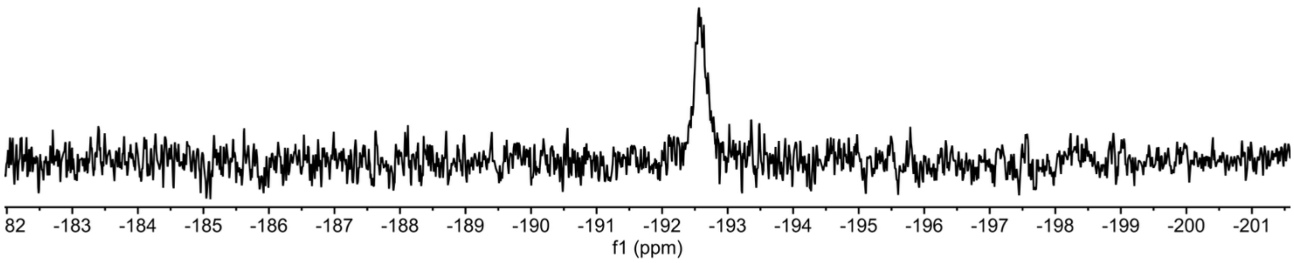

Supplementary Figure 18. $^{29}Si\{^{1}H\}$ NMR (79 MHz, C_6D_6, 298 K) spectrum of **4**.

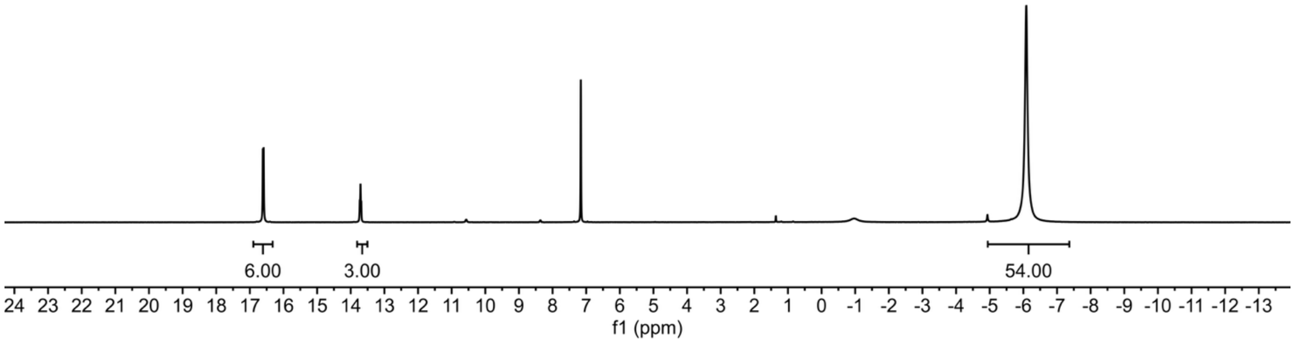

Supplementary Figure 19. ^{1}H NMR (400 MHz, C_6D_6, 298 K) spectrum of **5**.

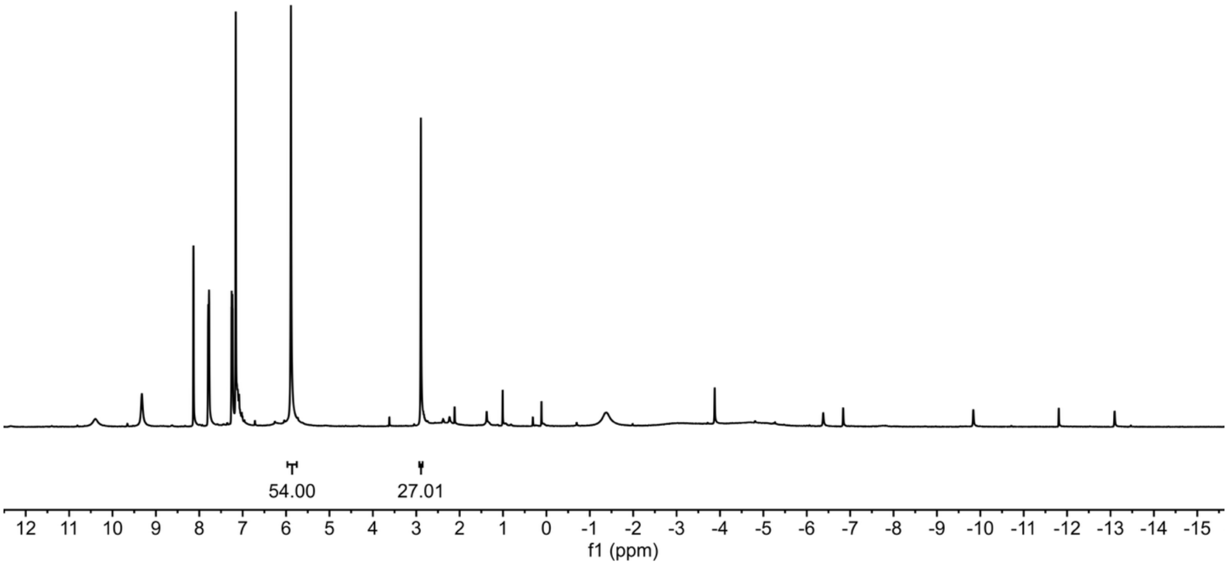

Supplementary Figure 20. ^{1}H NMR spectrum of the crude product from the reaction of **2** and **3** highlighting the 2:1 ratio of **8** and **7**.

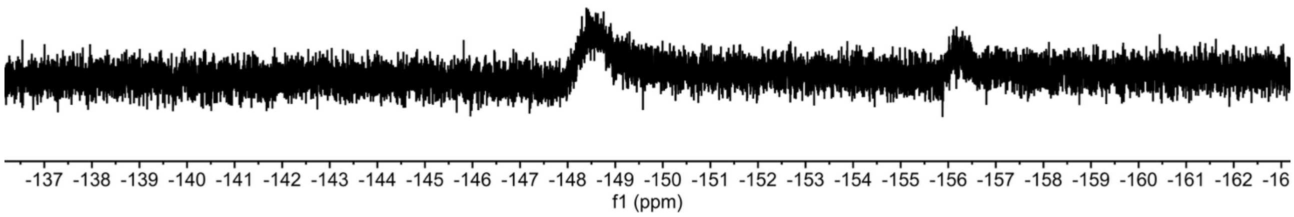

Supplementary Figure 21. ^{1}H NMR spectrum of the crude product from the reaction of **2** and **3** highlighting the *H*PP*H* resonance of **8** at ~ −147.5 ppm and the P*H* resonance of **7** at ~ −154.1 ppm.

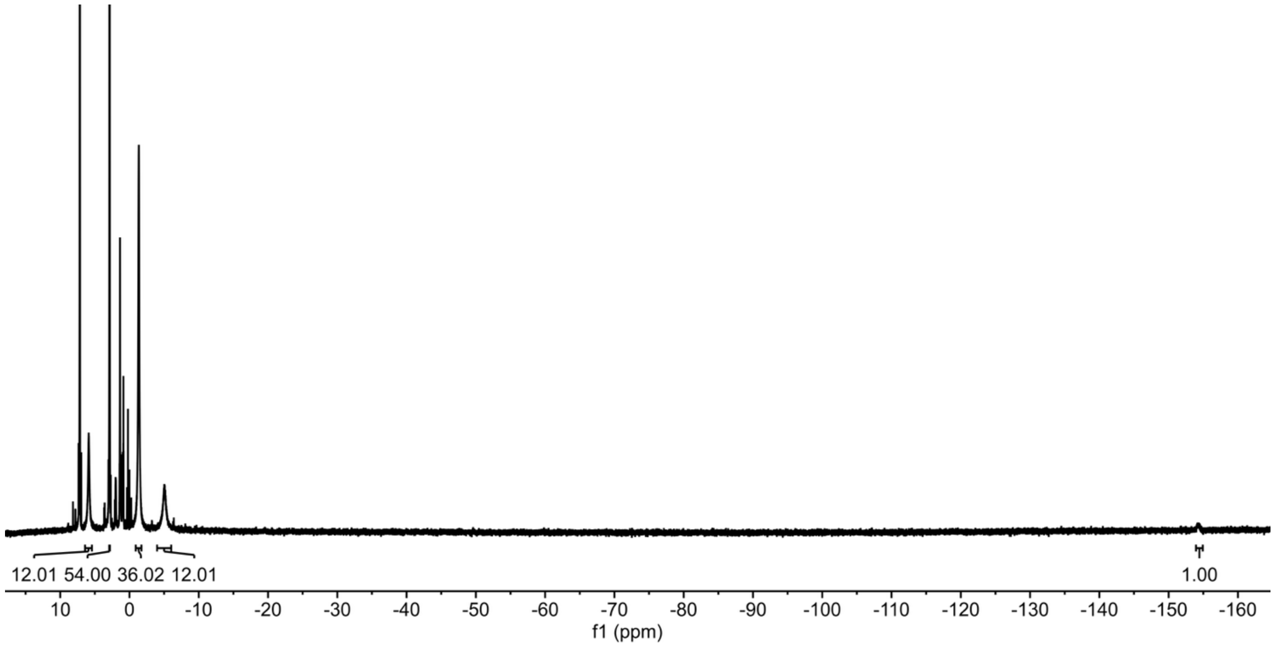

Supplementary Figure 22. ^{1}H NMR (400 MHz, C_6D_6, 298 K) spectrum of **7**.

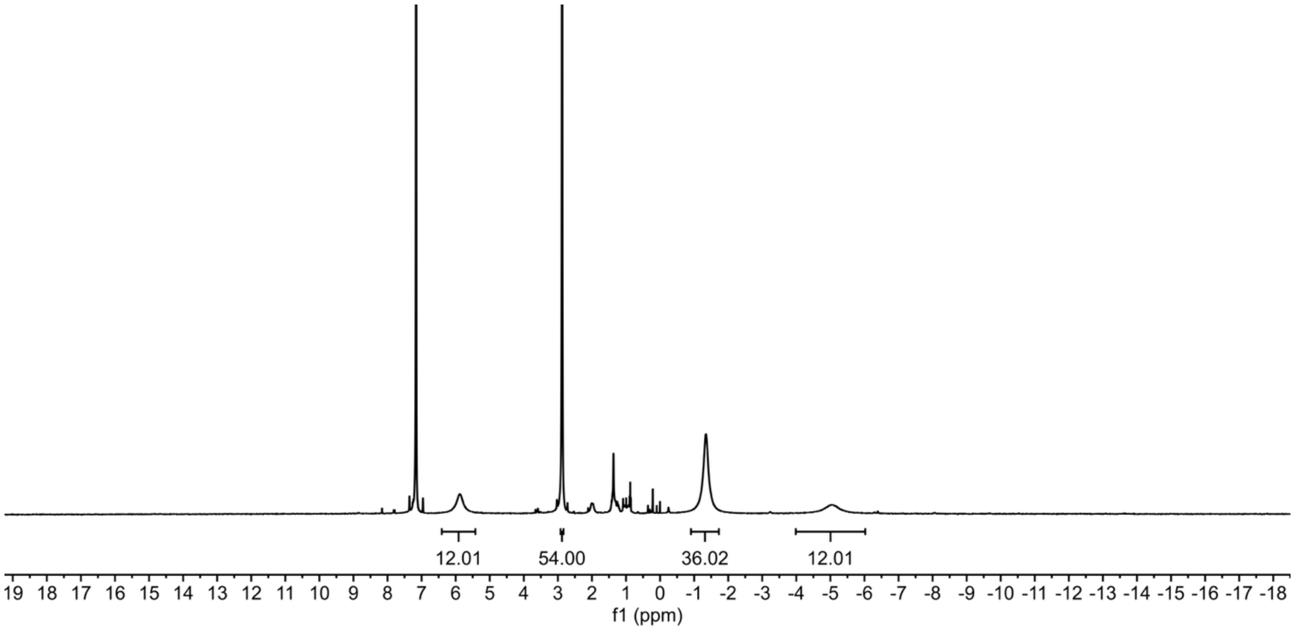

Supplementary Figure 23. Zoom-in of ^{1}H NMR (400 MHz, C_6D_6, 298 K) spectrum of **7**.

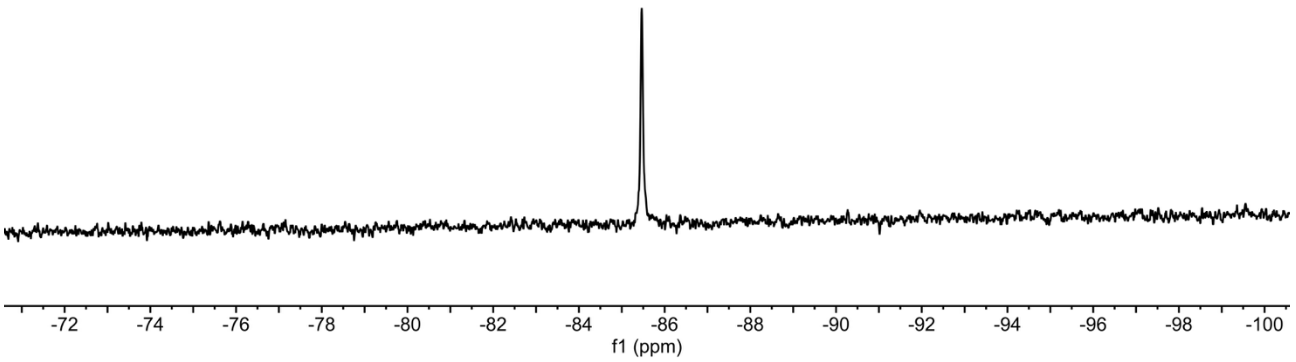

Supplementary Figure 24. $^{29}Si\{^{1}H\}$ NMR (79 MHz, C_6D_6, 298 K) spectrum of **7**.

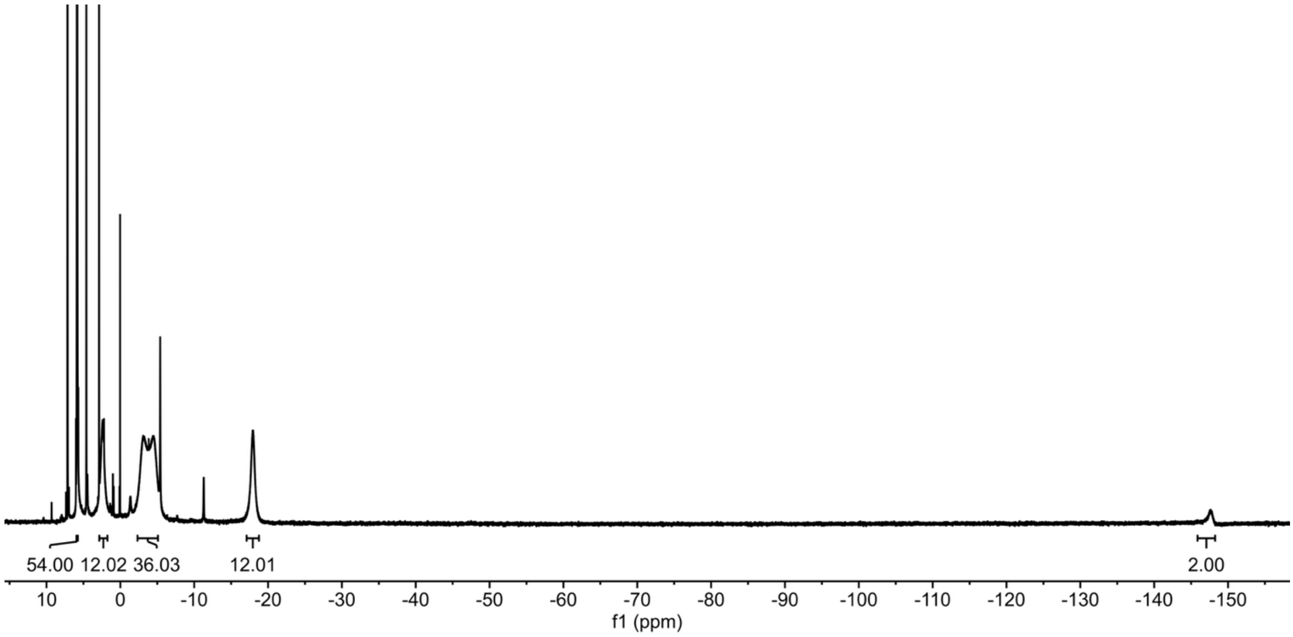

Supplementary Figure 25. ^{1}H NMR (400 MHz, C_6D_6, 298 K) spectrum of **8**.

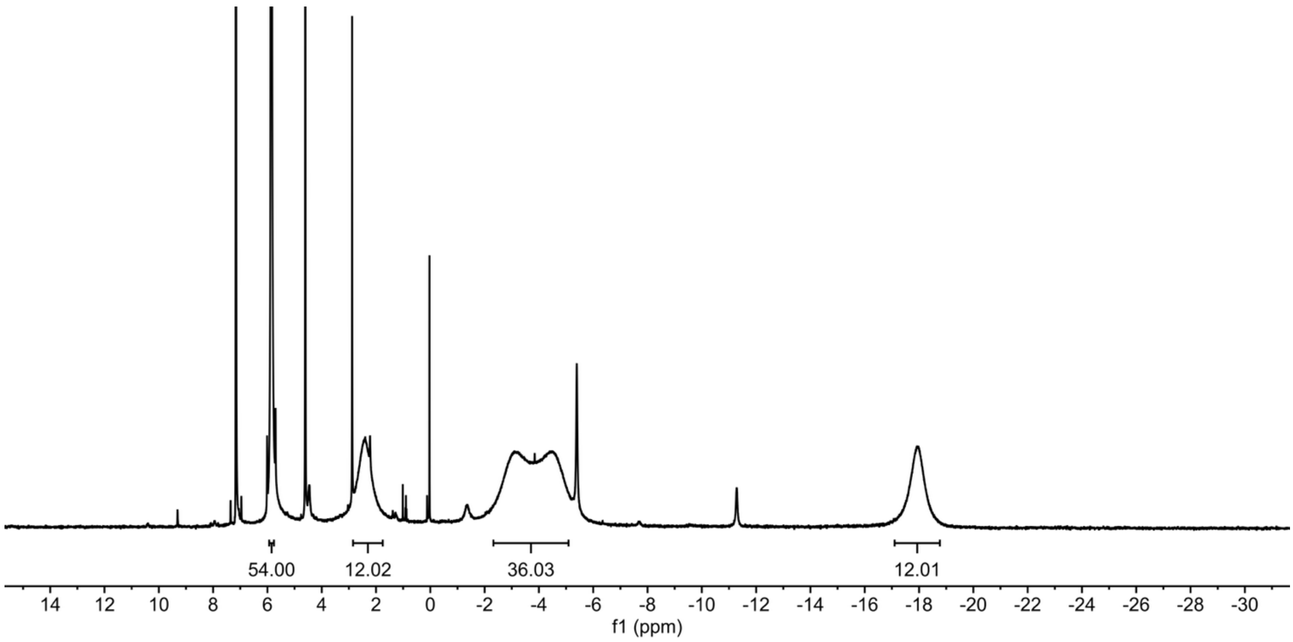

Supplementary Figure 26. Zoom-in of ^{1}H NMR (400 MHz, C_6D_6, 298 K) spectrum of **8**.

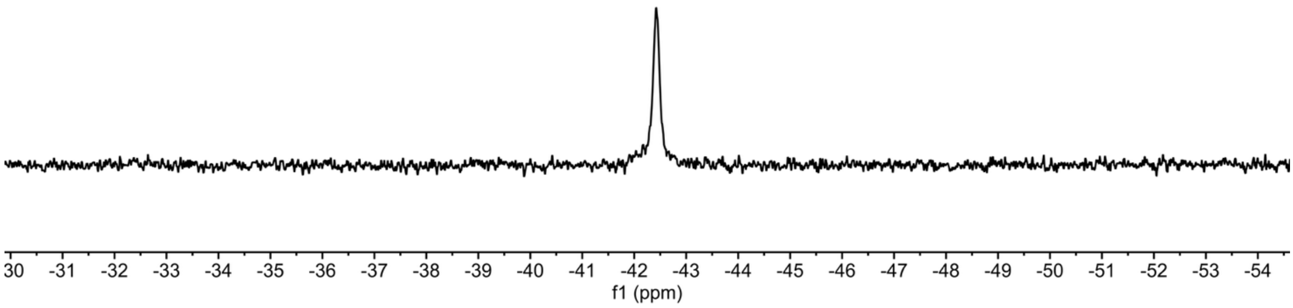

Supplementary Figure 27. $^{29}Si\{^1H\}$ NMR (79 MHz, C_6D_6, 298 K) spectrum of **8**.

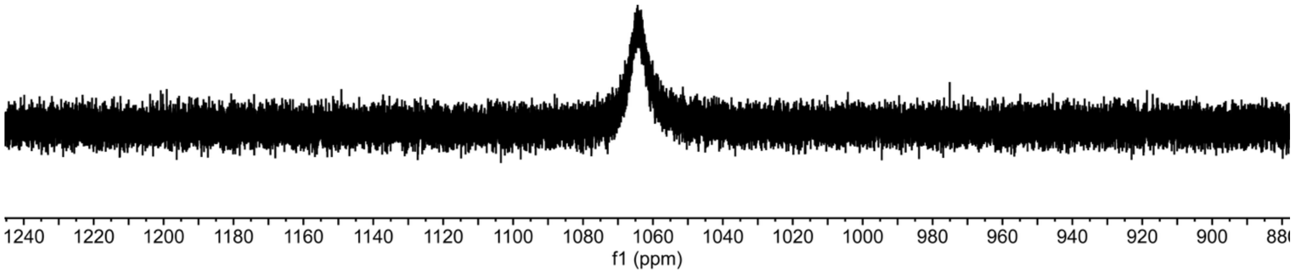

Supplementary Figure 28. ^{31}P NMR (165 MHz, C_6D_6, 298 K) spectrum of **8**.

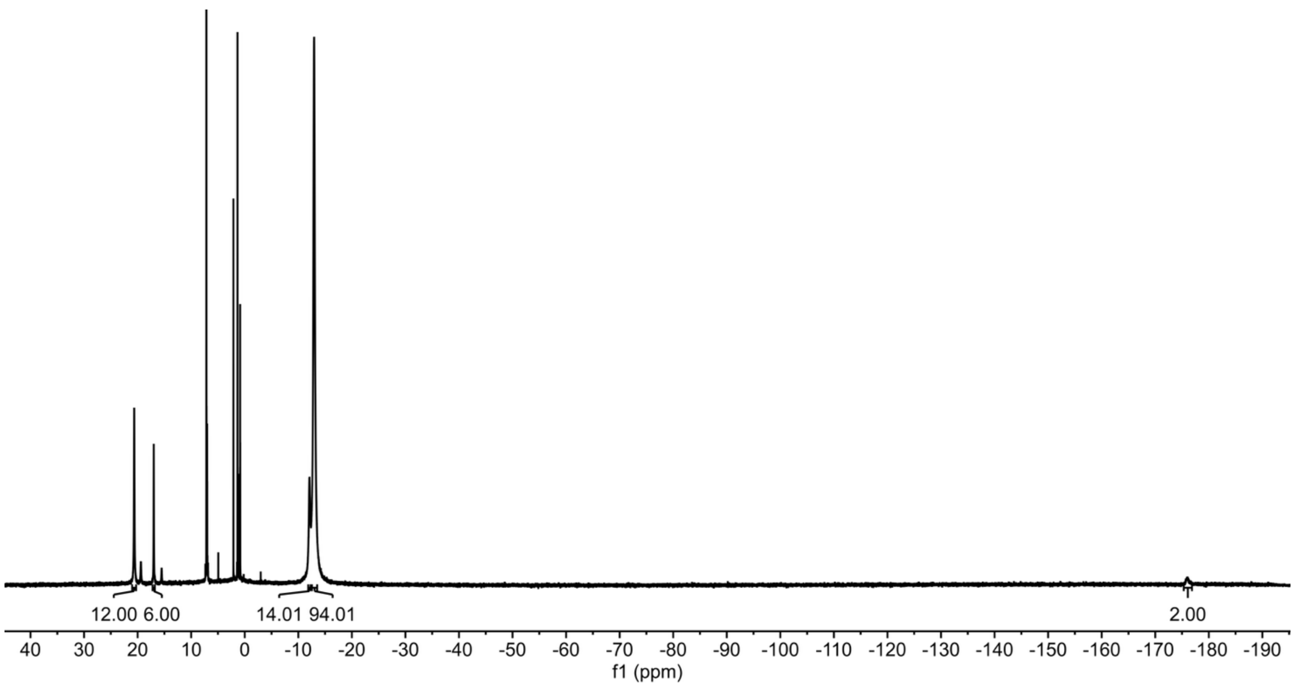

Supplementary Figure 29. ^{1}H NMR (400 MHz, C_6D_6, 298 K) spectrum of **9**.

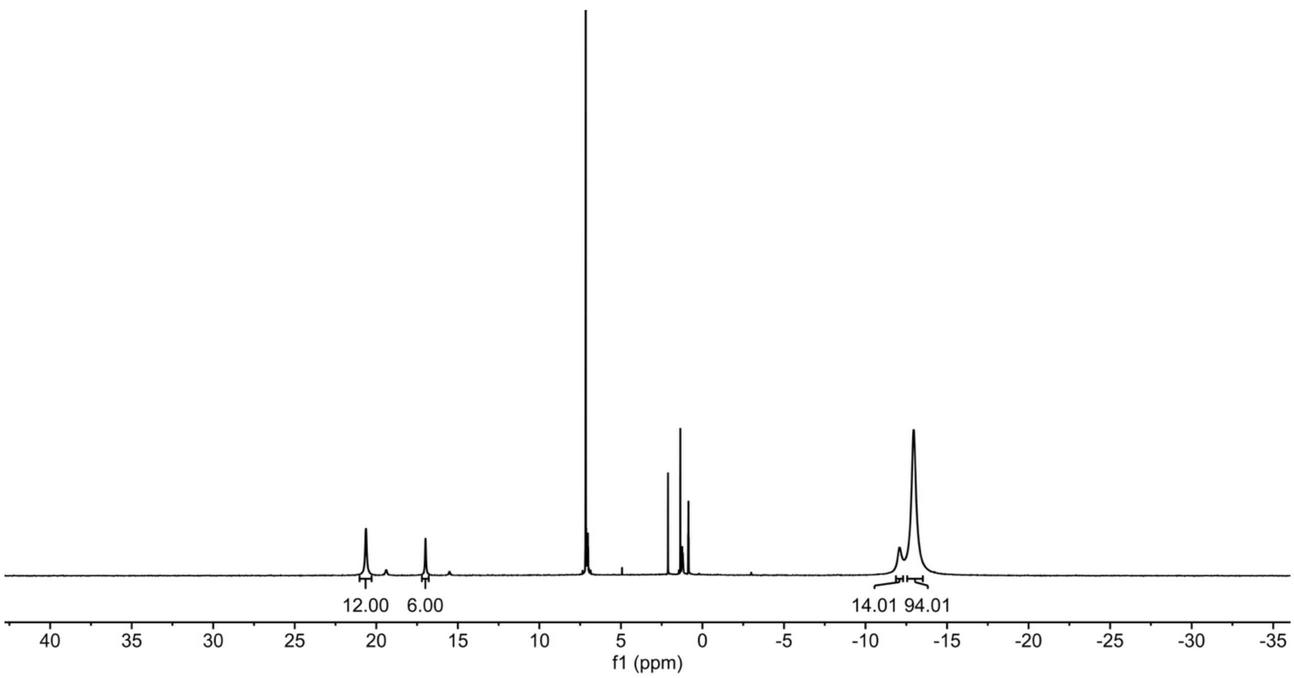

Supplementary Figure 30. Zoom-in of ^{1}H NMR (400 MHz, C_6D_6, 298 K) spectrum of **9**.

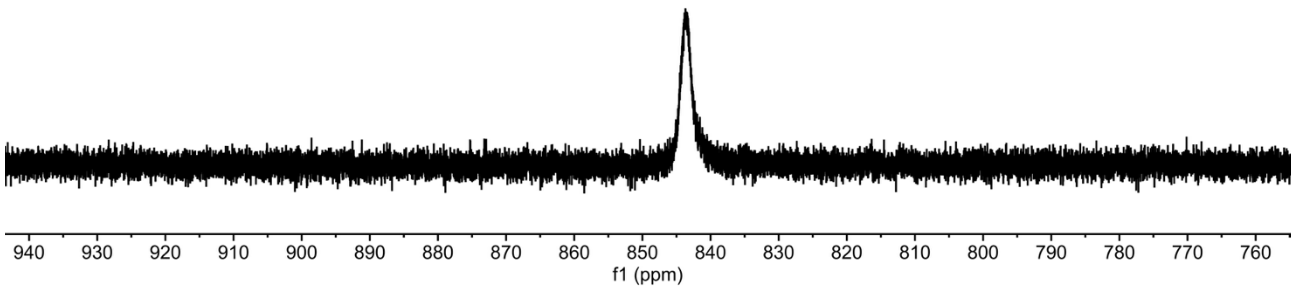

Supplementary Figure 31. ^{31}P NMR (165 MHz, C_6D_6, 298 K) spectrum of **9**.

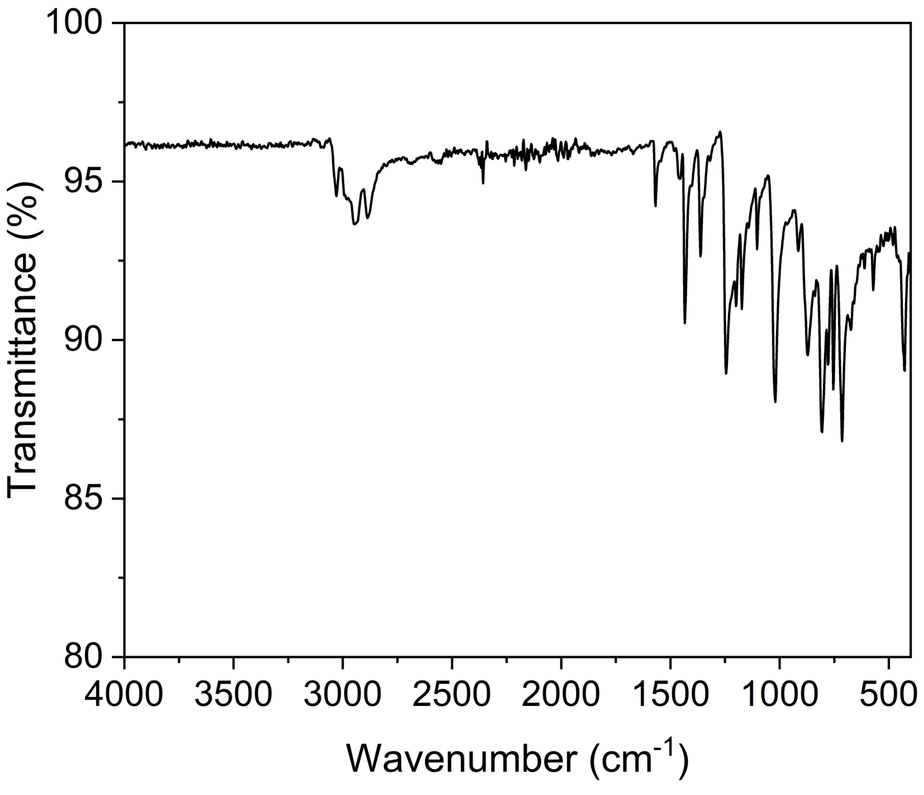

Supplementary Figure 32. ATR-IR spectrum of [(Anthracene)Mg(THF)$_3$].

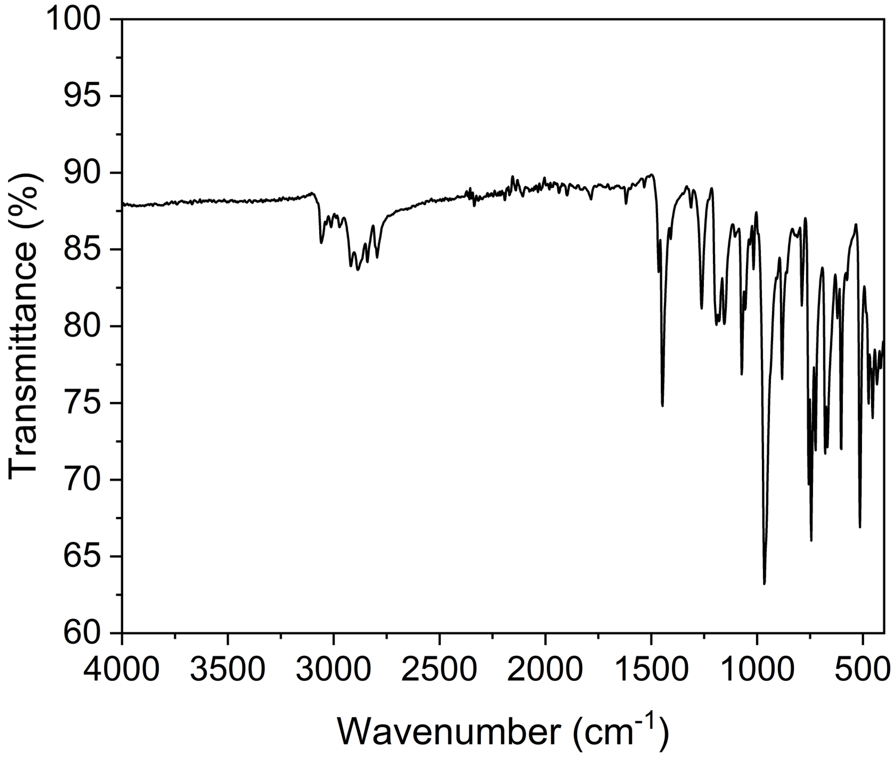

Supplementary Figure 33. ATR-IR spectrum of **1**.

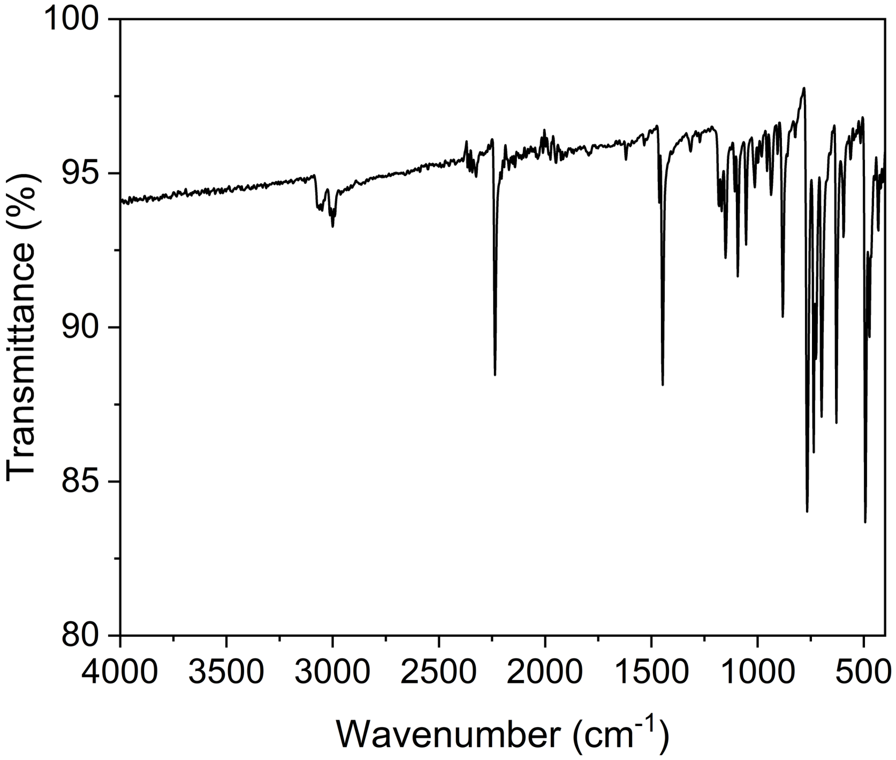

Supplementary Figure 34. ATR-IR spectrum of **2**.

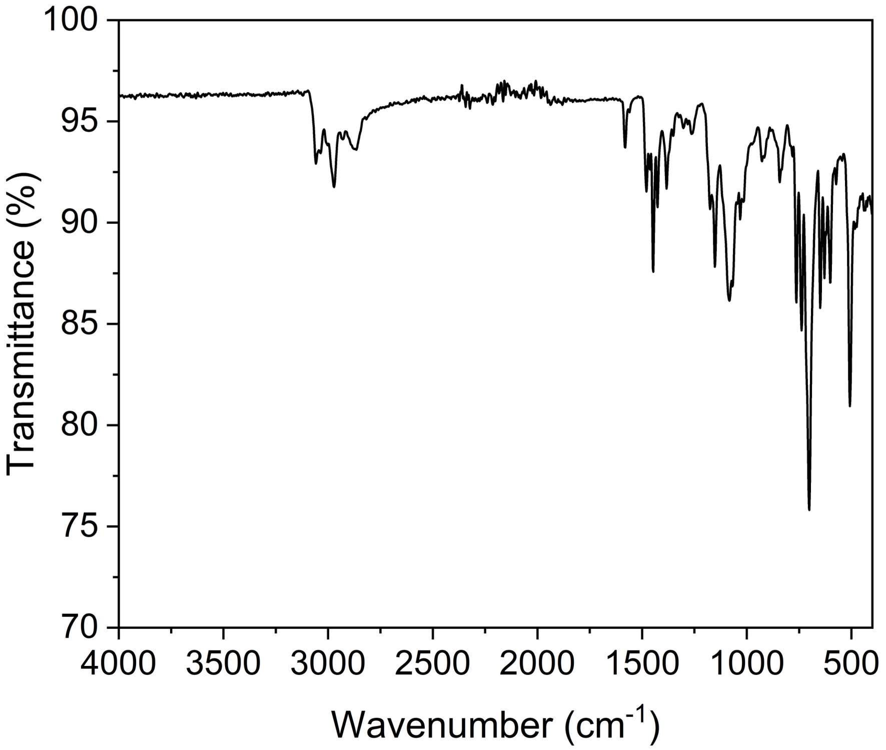

Supplementary Figure 35. ATR-IR spectrum of PDBN-(BPh_3)Na(OEt_2)$_2$.

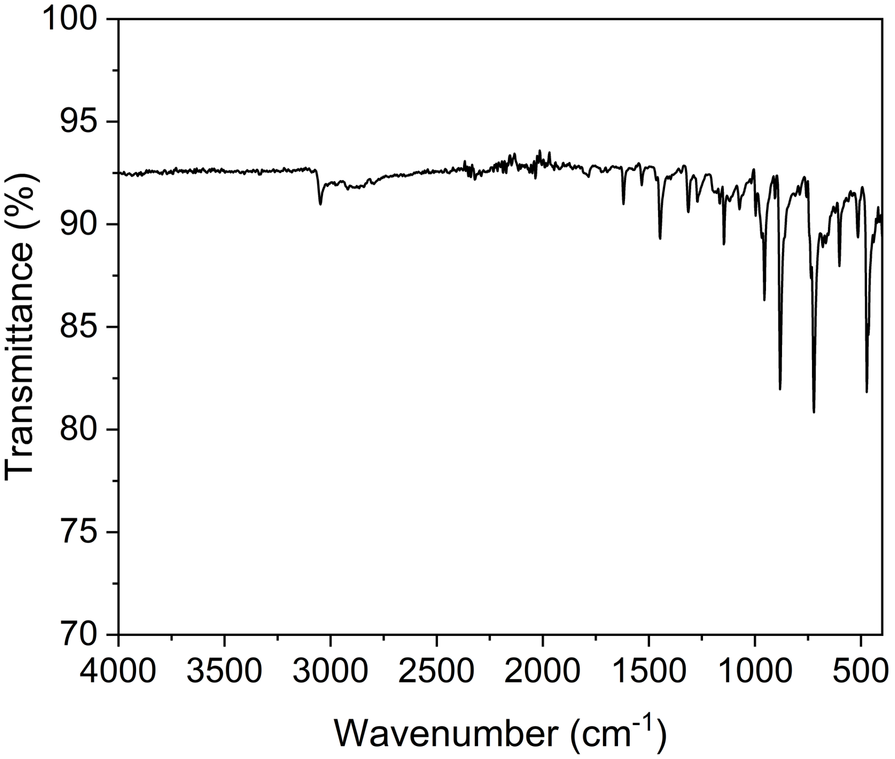

Supplementary Figure 36. ATR-IR spectrum of PDBN-Bu^t.

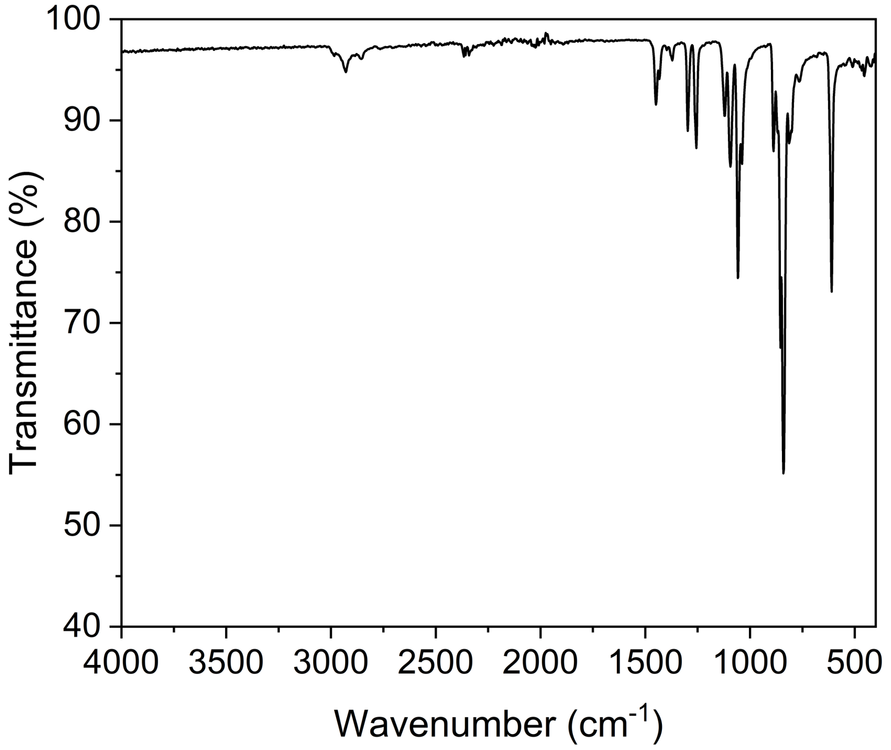

Supplementary Figure 37. ATR-IR spectrum of UI_3(1,4-dioxane)$_{1.5}$.

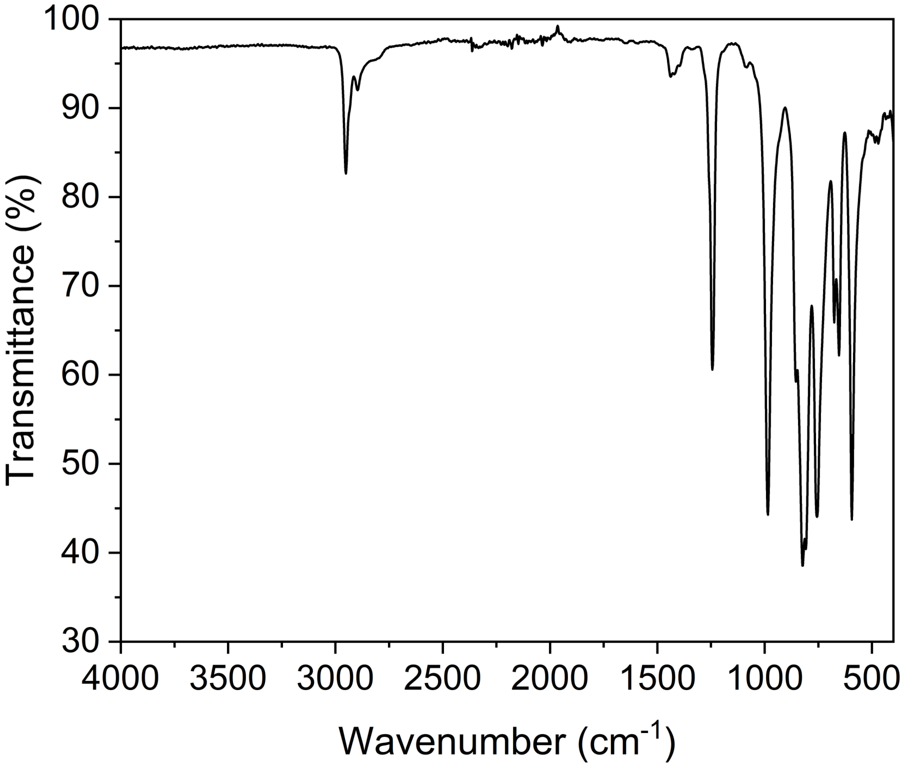

Supplementary Figure 38. ATR-IR spectrum of [U{N($SiMe_3$)$_2$}$_3$].

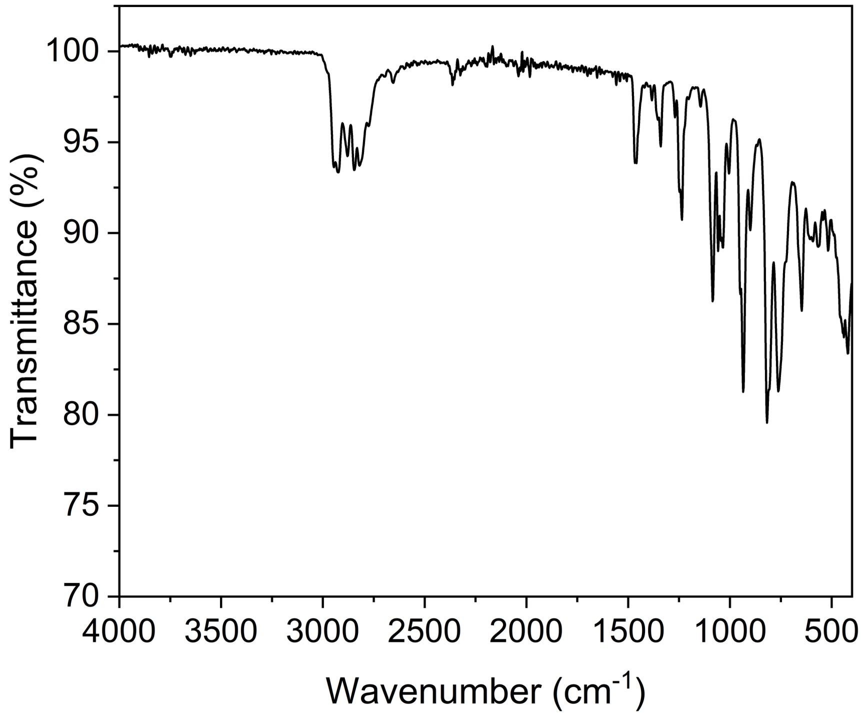

Supplementary Figure 39. ATR-IR spectrum of $Tren^{DMBS}Li_3(THF)_3$.

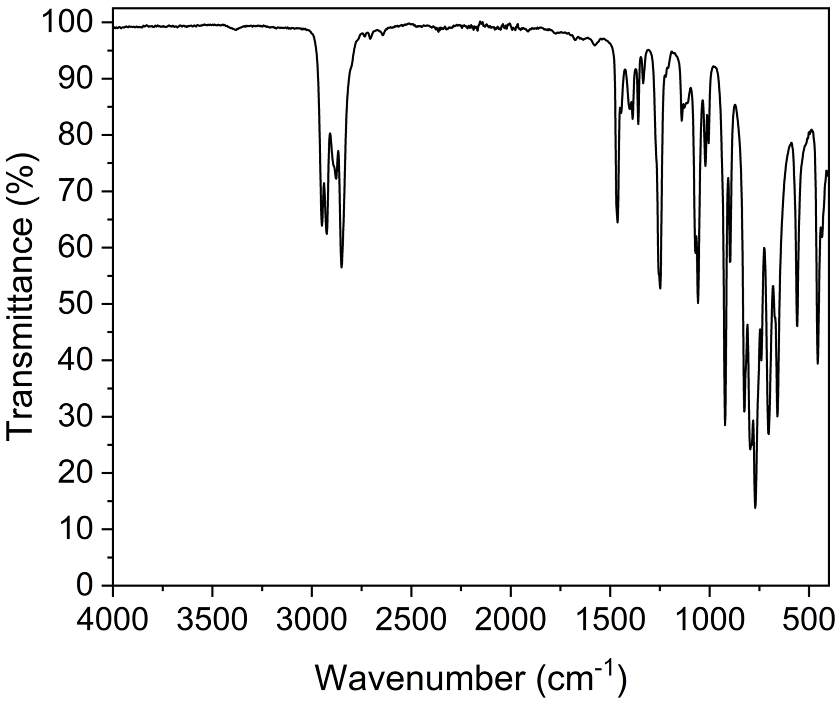

Supplementary Figure 40. ATR-IR spectrum of $[(Tren^{DMBS})U^{IV}(Cl)]$.

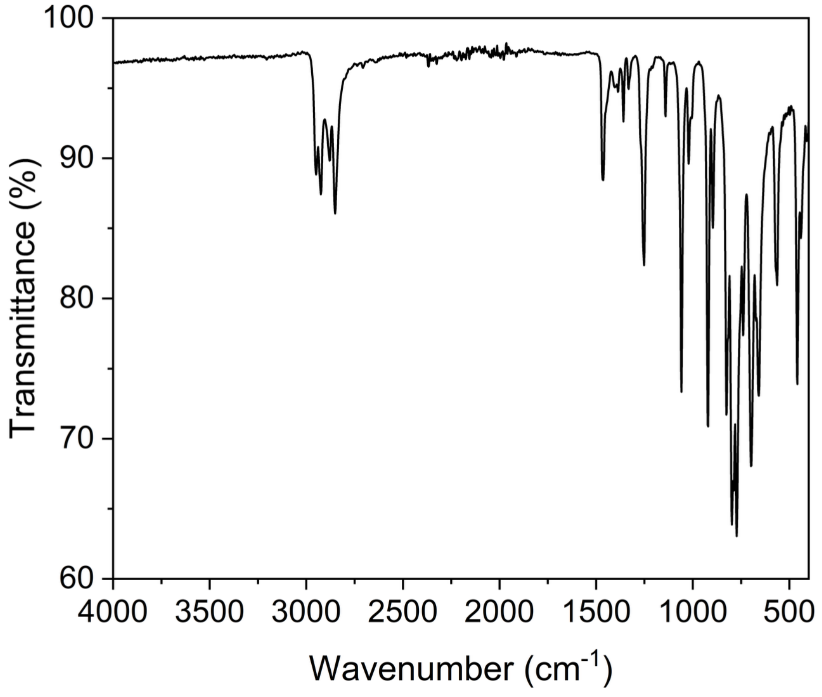

Supplementary Figure 41. ATR-IR spectrum of [(Tren^{DMBS})U^{IV}(I)].

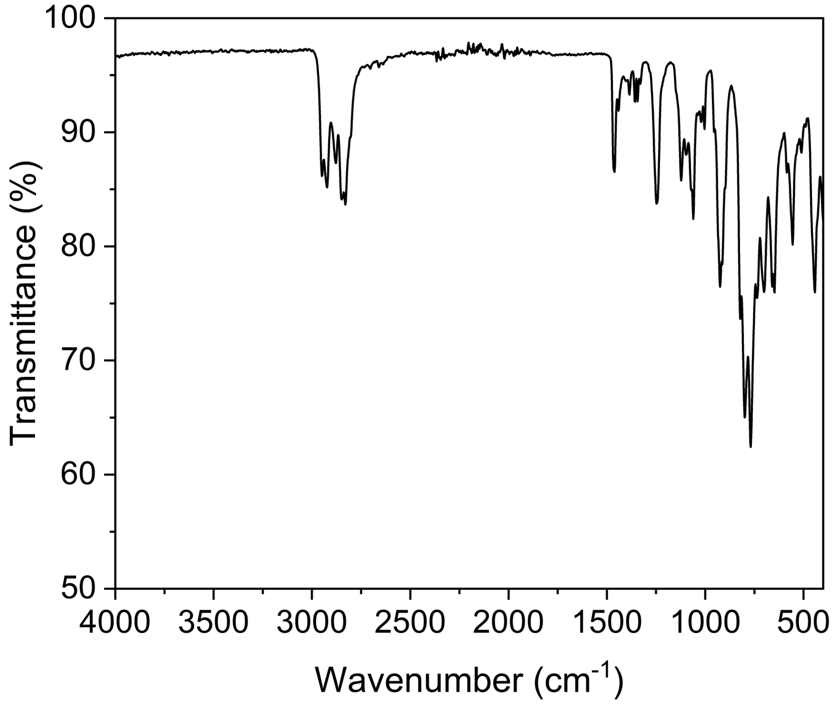

Supplementary Figure 42. ATR-IR spectrum of **3**.

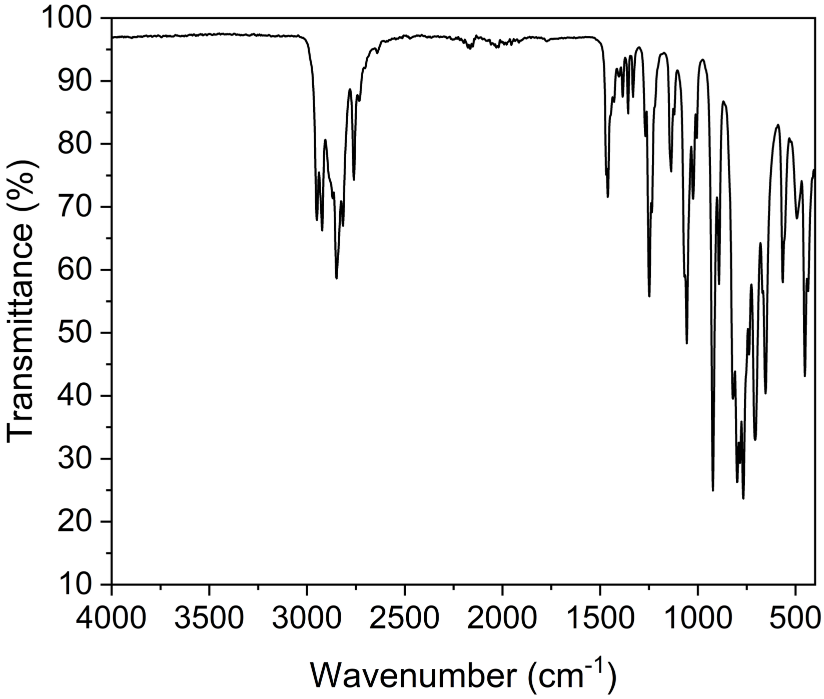

Supplementary Figure 43. ATR-IR spectrum of **4**.

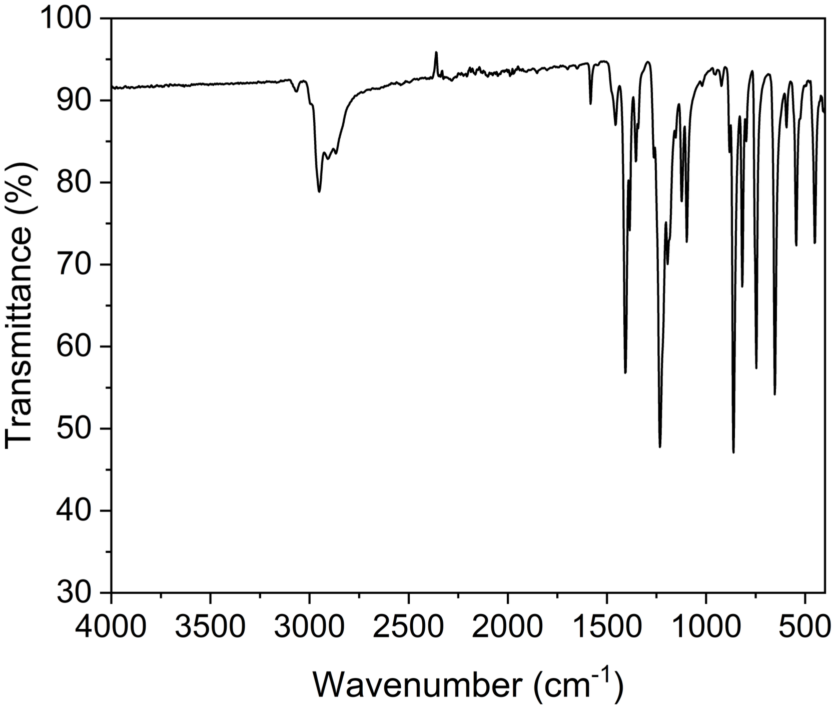

Supplementary Figure 44. ATR-IR spectrum of **5**.

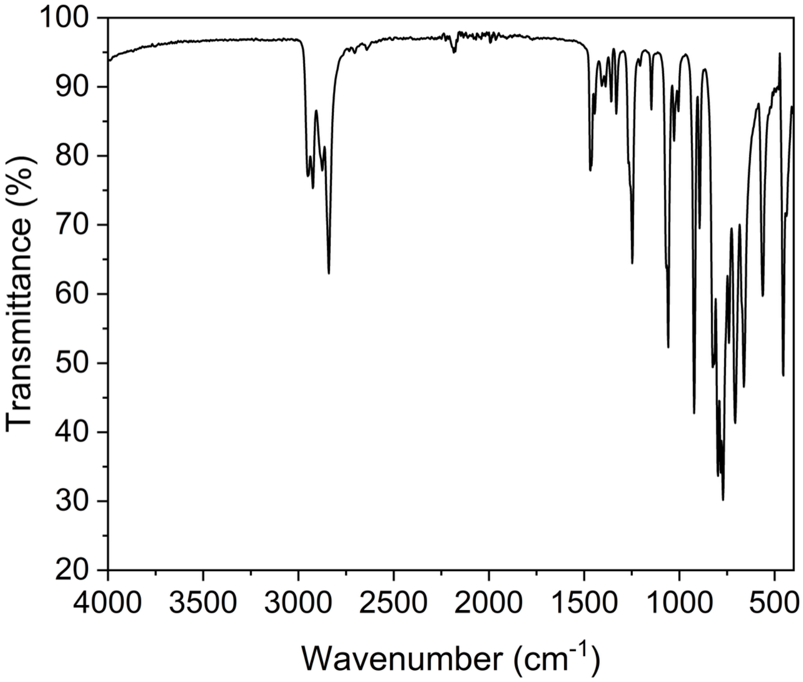

Supplementary Figure 45. ATR-IR spectrum of **7**.

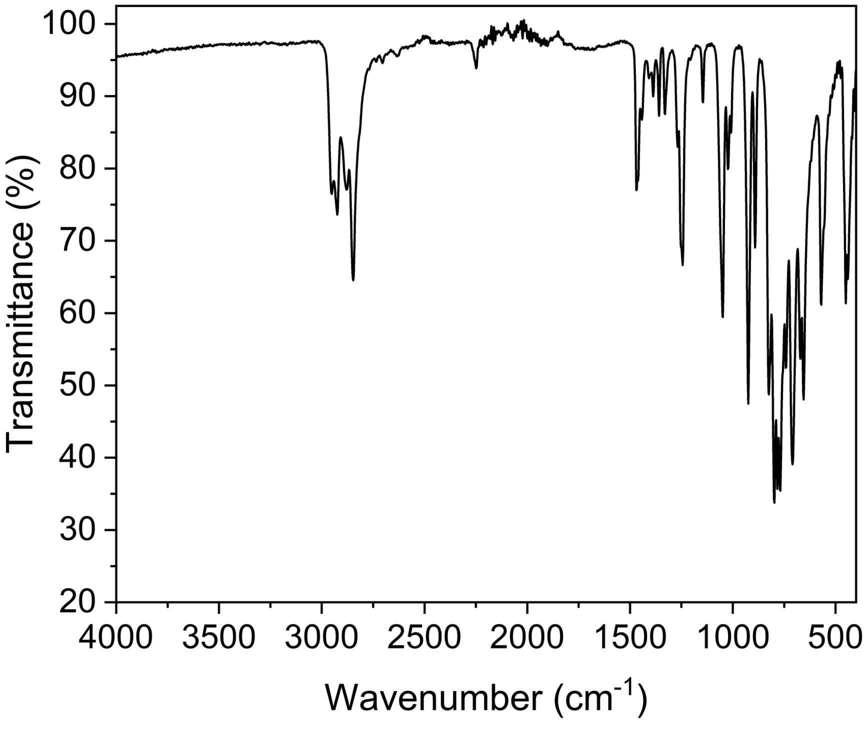

Supplementary Figure 46. ATR-IR spectrum of **8**.

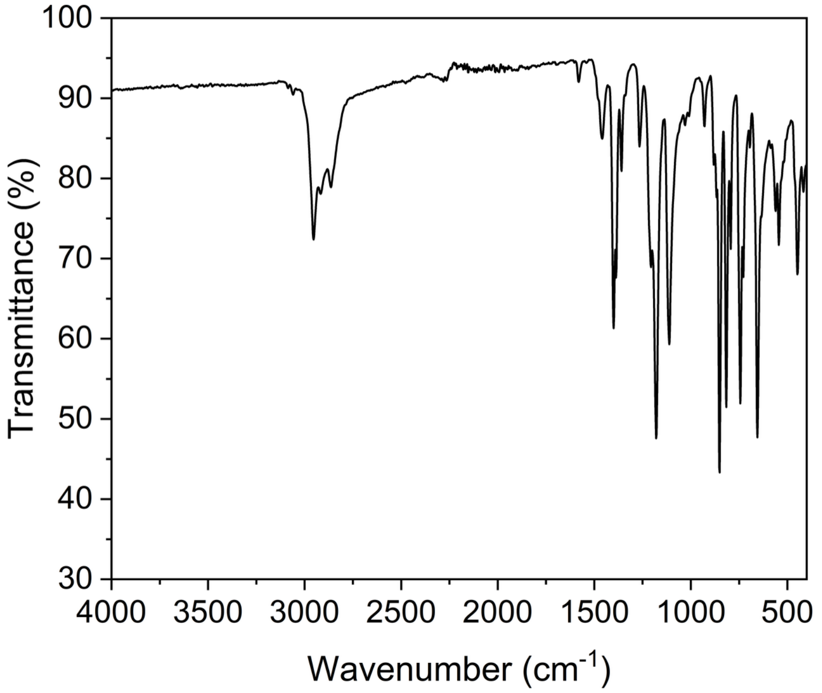

Supplementary Figure 47. ATR-IR spectrum of **9**.

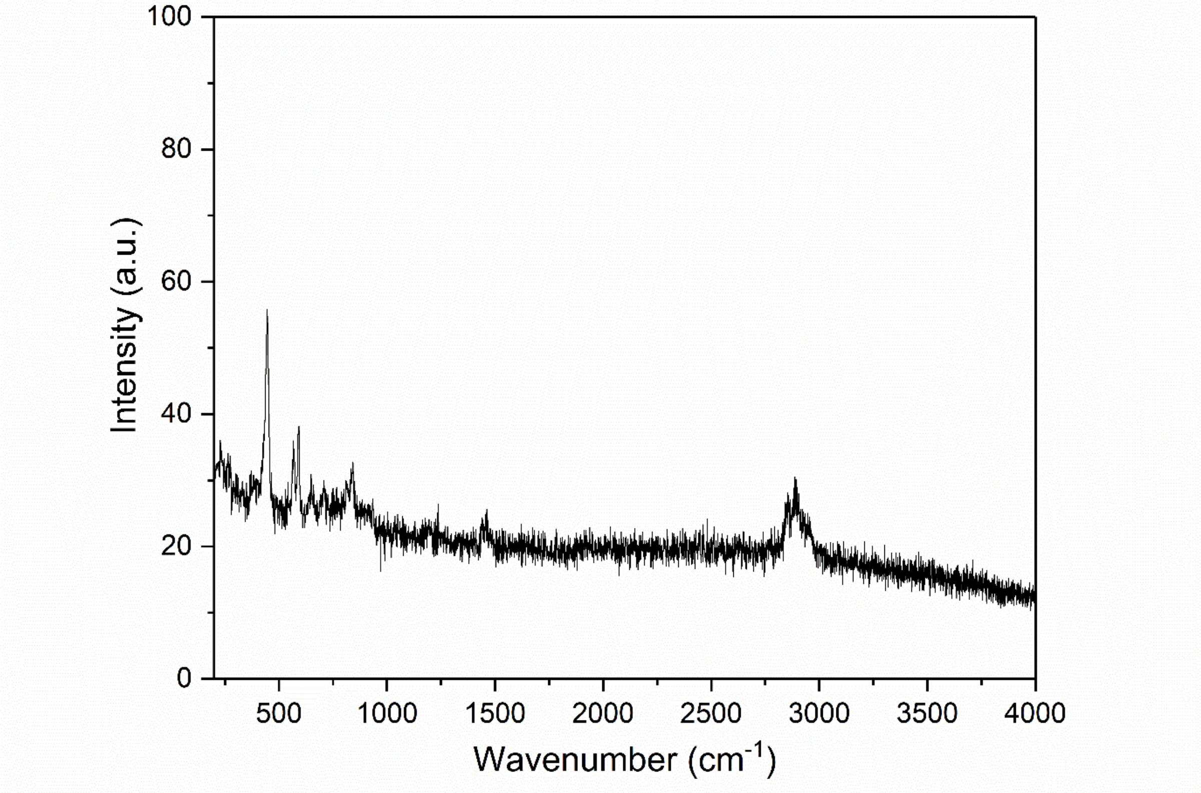

Supplementary Figure 48. Raman spectrum of crystalline **8** (200 to 4000 cm^{-1}).

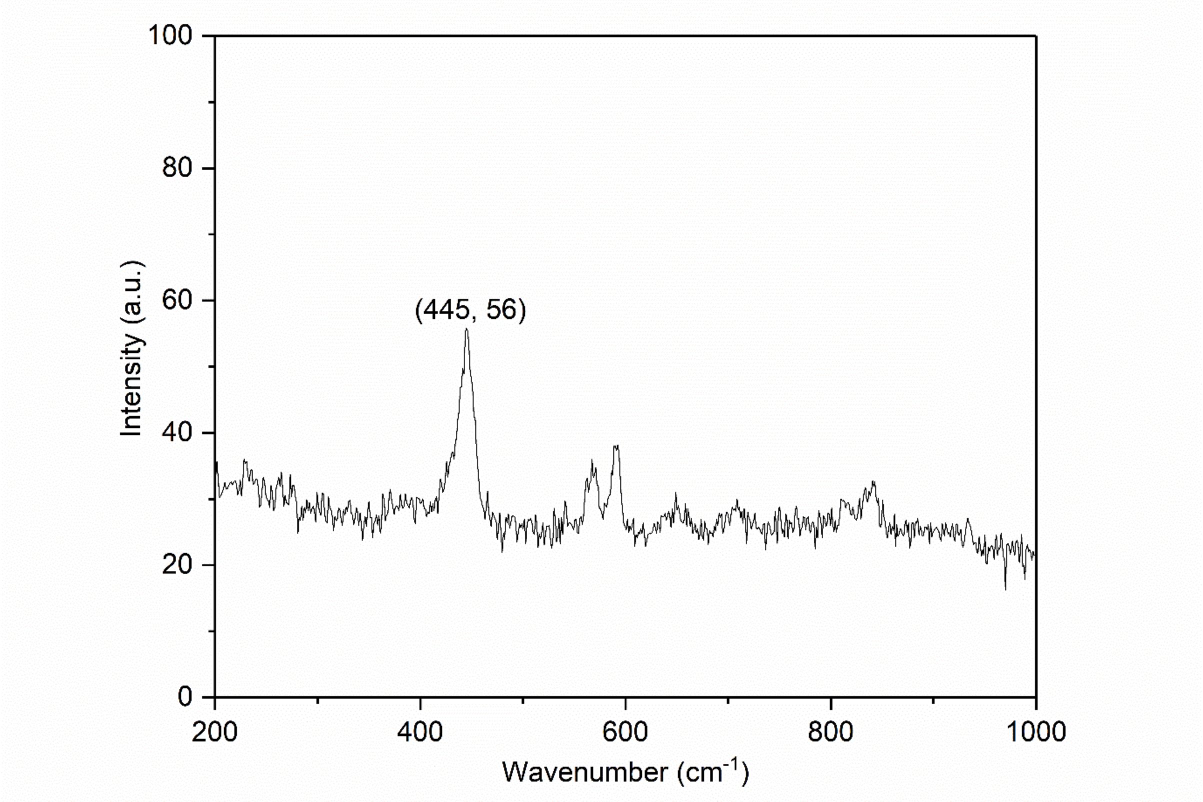

Supplementary Figure 49. Zoom-in of the Raman spectrum of crystalline **8** (200 to 1000 cm^{-1}).

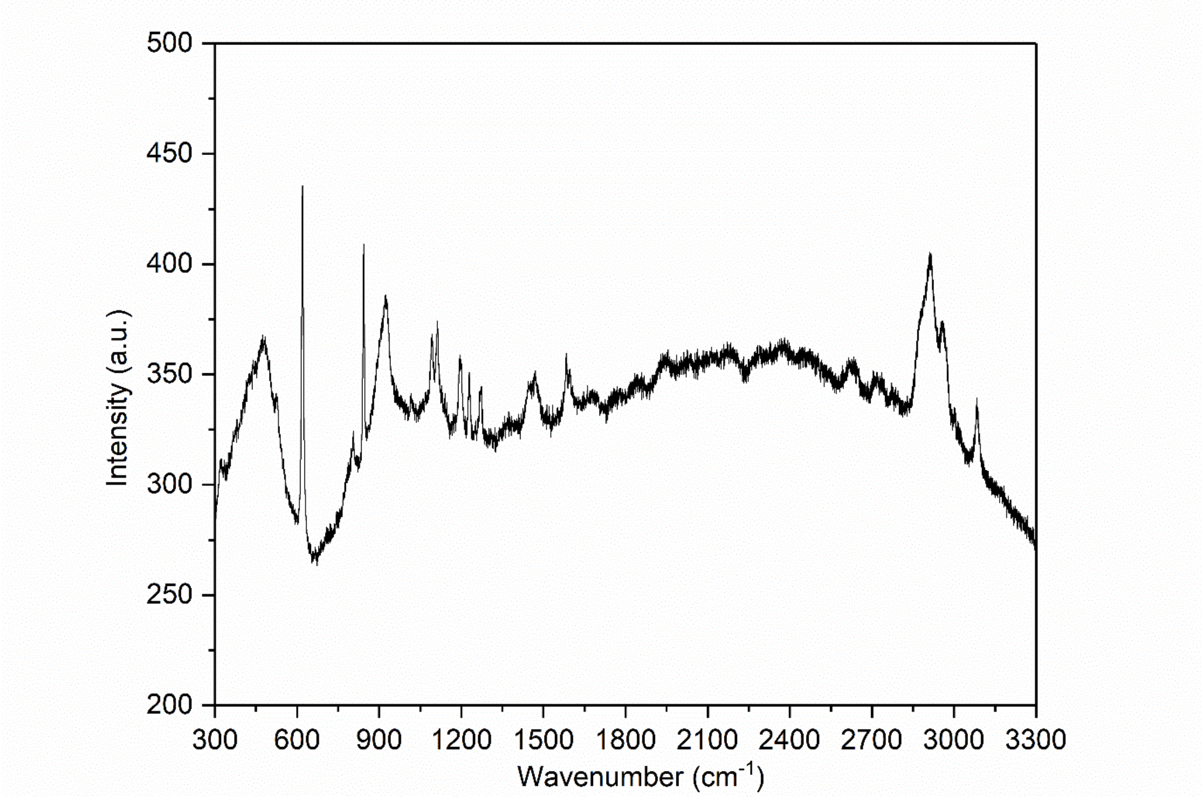

Supplementary Figure 50. Raman spectrum of crystalline **9** (300 to 3300 cm^{-1}).

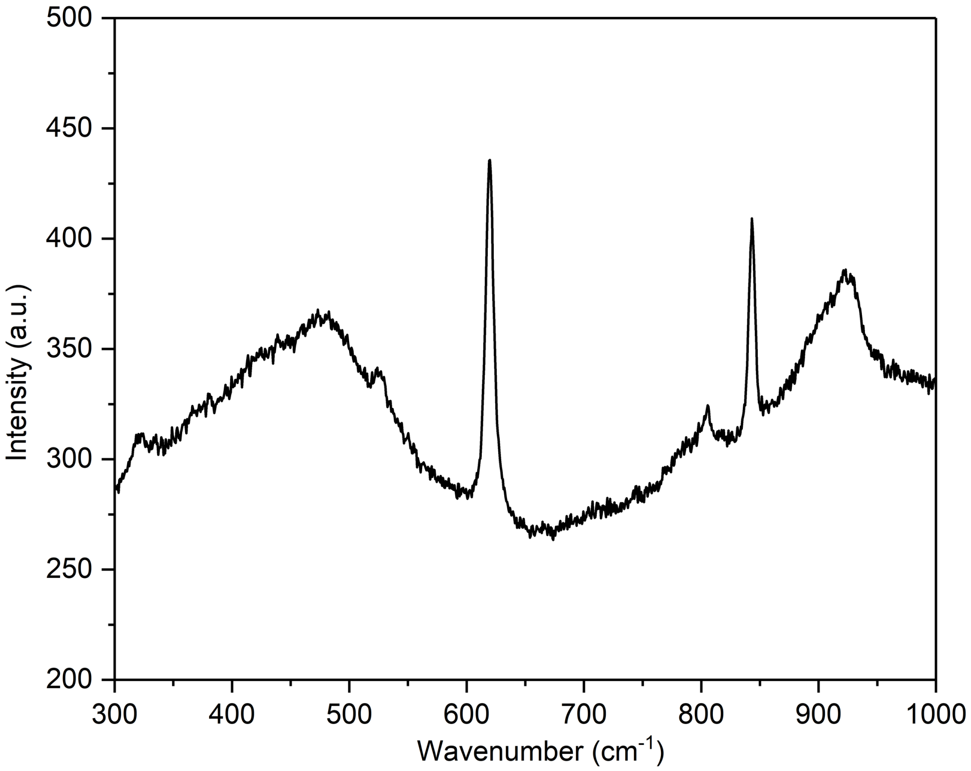

Supplementary Figure 51. Zoom-in of the Raman spectrum of crystalline **9** (300 to 1000 cm^{-1}).

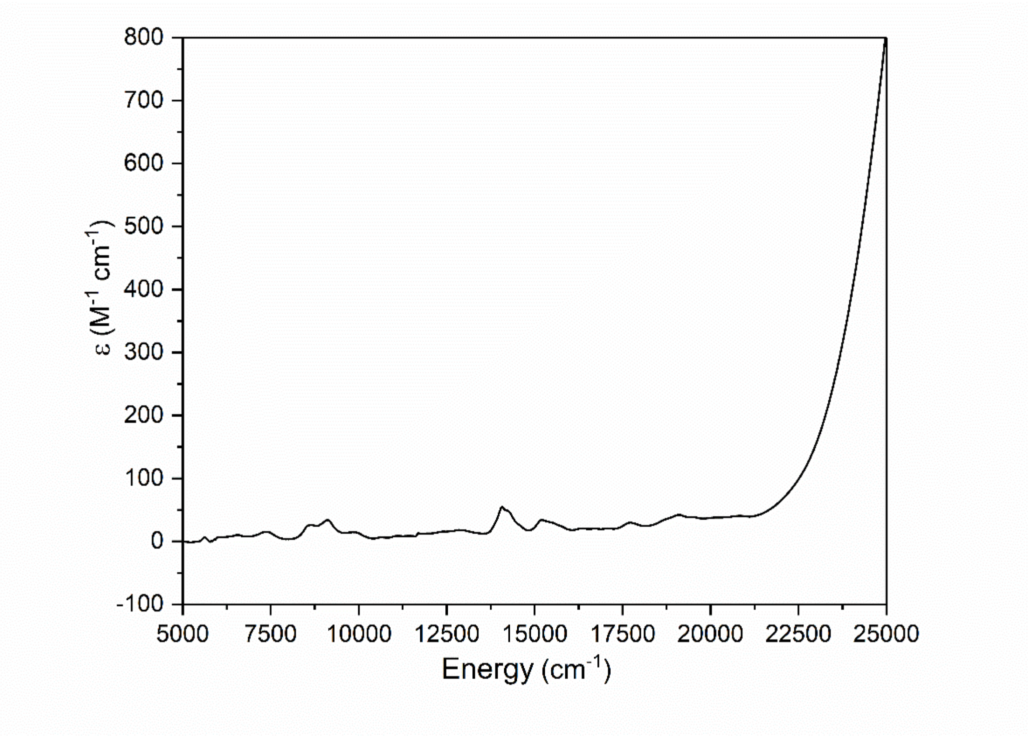

Supplementary Figure 52. UV/Vis/NIR spectrum of **4** (25 mM) in THF over the range 5000-25000 cm^{-1}.

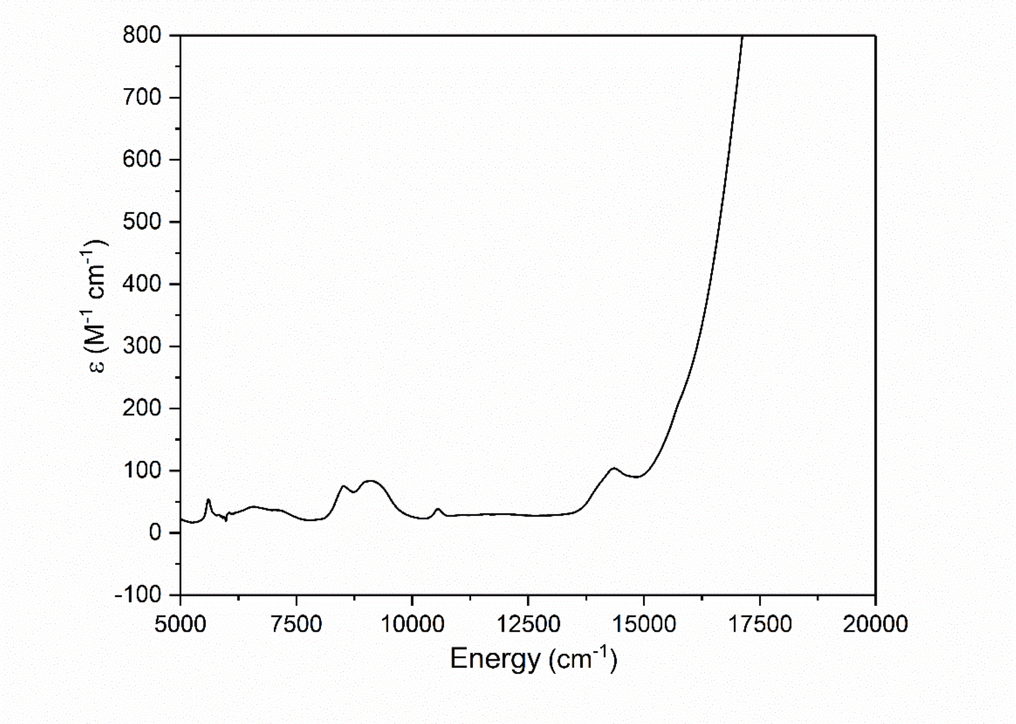

Supplementary Figure 53. UV/Vis/NIR spectrum of **8** (25 mM) in benzene over the range 5000-20000 cm^{-1}.

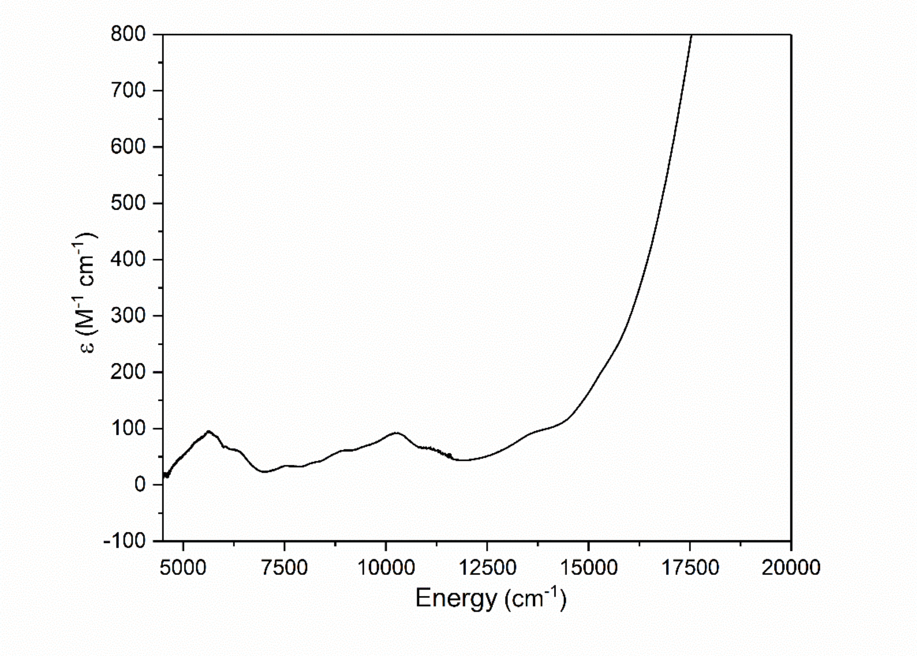

Supplementary Figure 54. UV/Vis/NIR spectrum of **9** (25 mM) in benzene over the range 5000-20000 cm^{-1}.

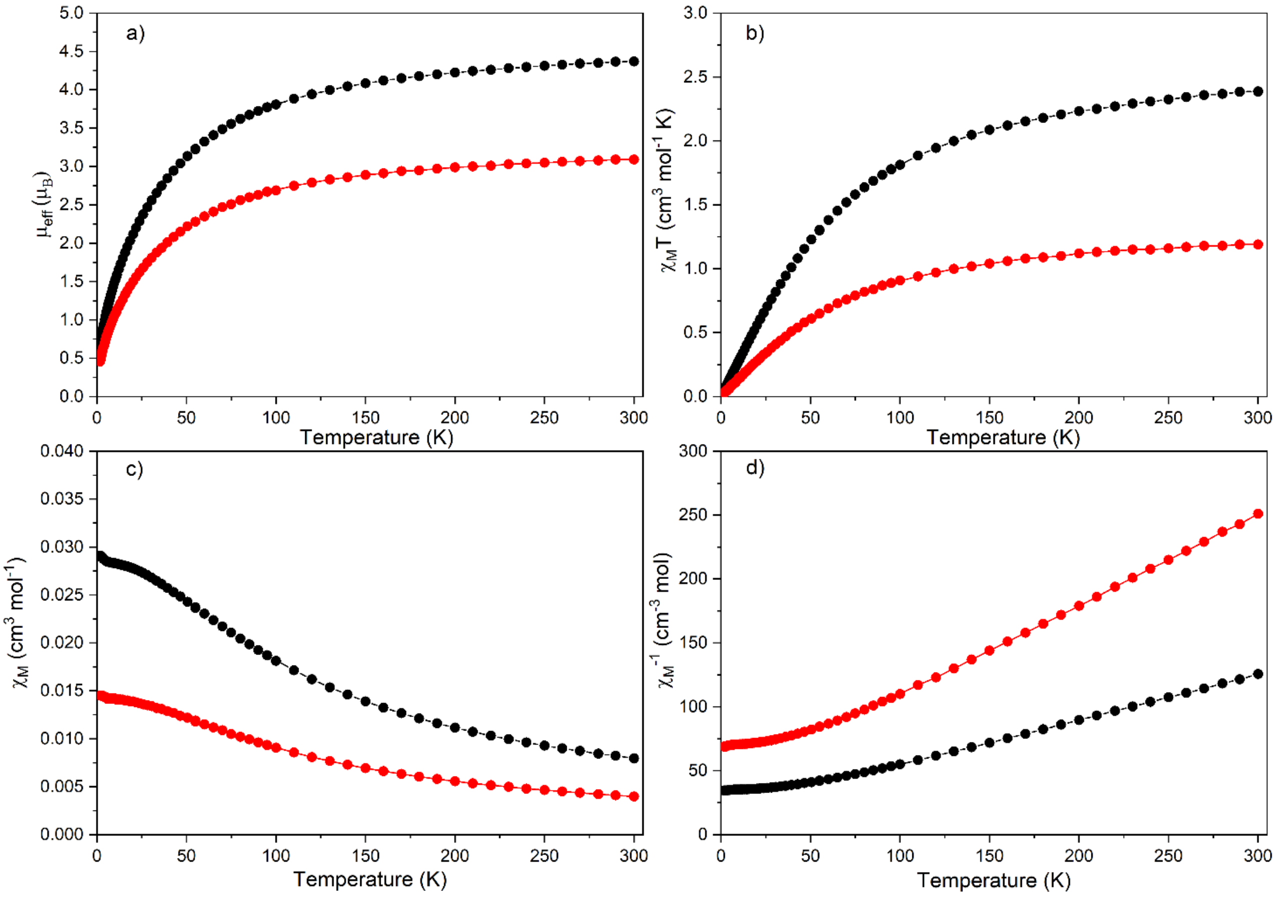

Supplementary Figure 55. Variable-temperature SQUID magnetometry of **8** (black, whole molecule; red, per U ion) over the temperature range 2-300 K: a) μ_{eff} vs T; b) $\chi_M T$ vs T; c) χ_M vs T; d) χ_M^{-1} vs T.

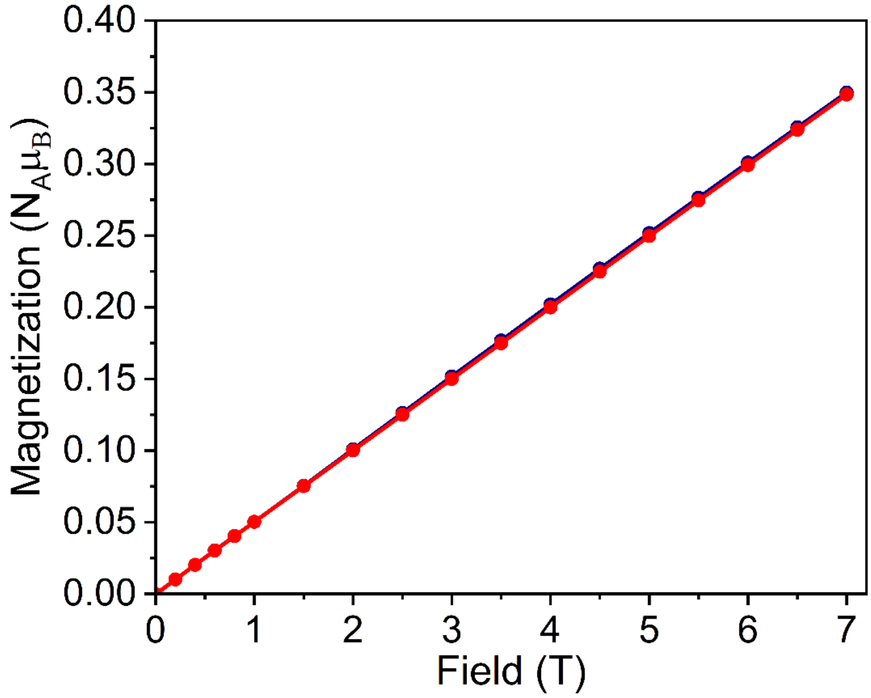

Supplementary Figure 56. Magnetization vs Field data for **8** at 2 and 4 K.

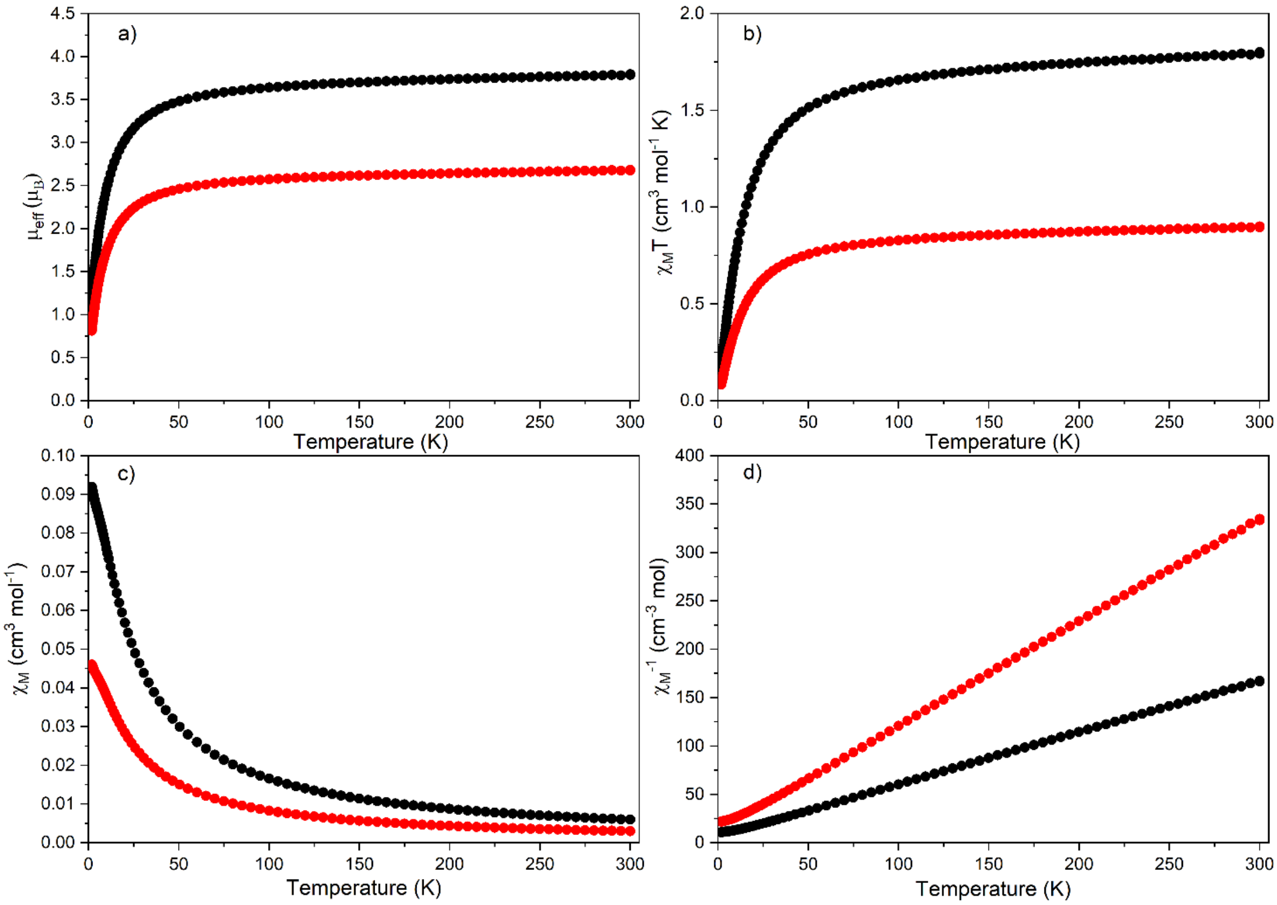

Supplementary Figure 57. Variable-temperature SQUID magnetometry of **9** (black, whole molecule; red, per U ion) over the temperature range 2-300 K: a) μ_{eff} vs T; b) $\chi_M T$ vs T; c) χ_M vs T; d) χ_M^{-1} vs T.

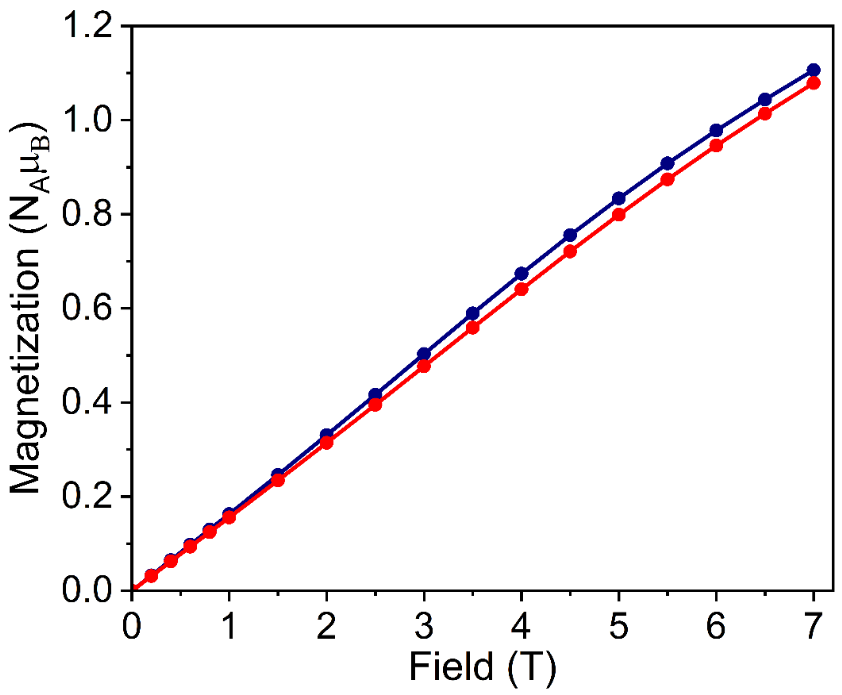

Supplementary Figure 58. Magnetization vs Field data for **9** at 2 and 4 K.

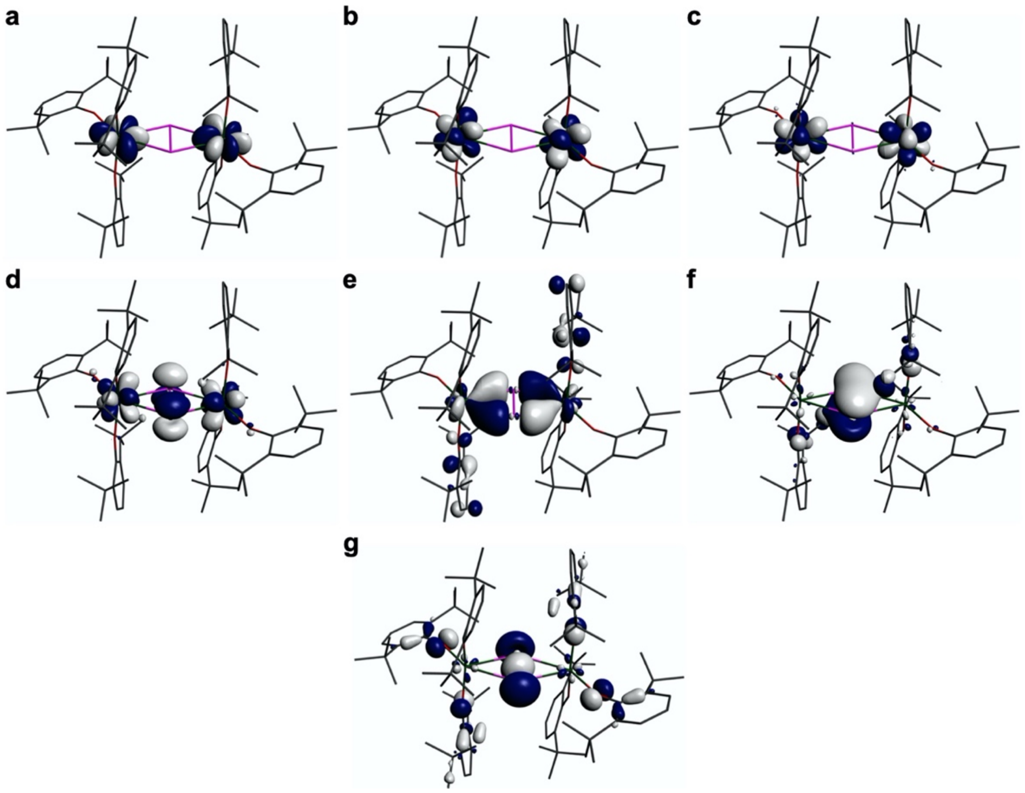

Supplementary Figure 59. Selected molecular orbitals of **6**. a) HOMO (448a, −3.599 eV), b) HOMO−1 (447a, −3.688 eV), c) HOMO−2 (446a, −3.707 eV), d) HOMO−3 (445a, −3.879 eV), e) HOMO−8 (440a, −5.186 eV), f) HOMO−17 (431a, −6.247 eV), g) HOMO−21 (427a, −6.552 eV).

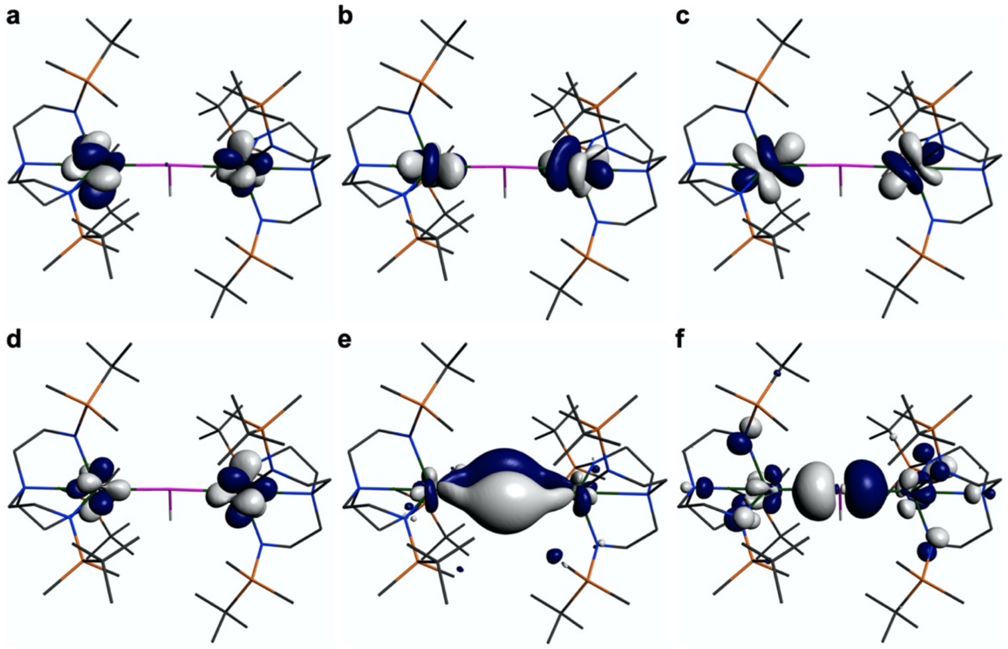

Supplementary Figure 60. Selected molecular orbitals of **7**. a) HOMO (373a, −2.753 eV), b) HOMO−1 (372a, −2.759 eV), c) HOMO−2 (371a, −2.774 eV), d) HOMO−3 (370a, −2.857 eV), e) HOMO−4 (369a, −3.954 eV), f) HOMO−5 (368a, −4.469 eV).

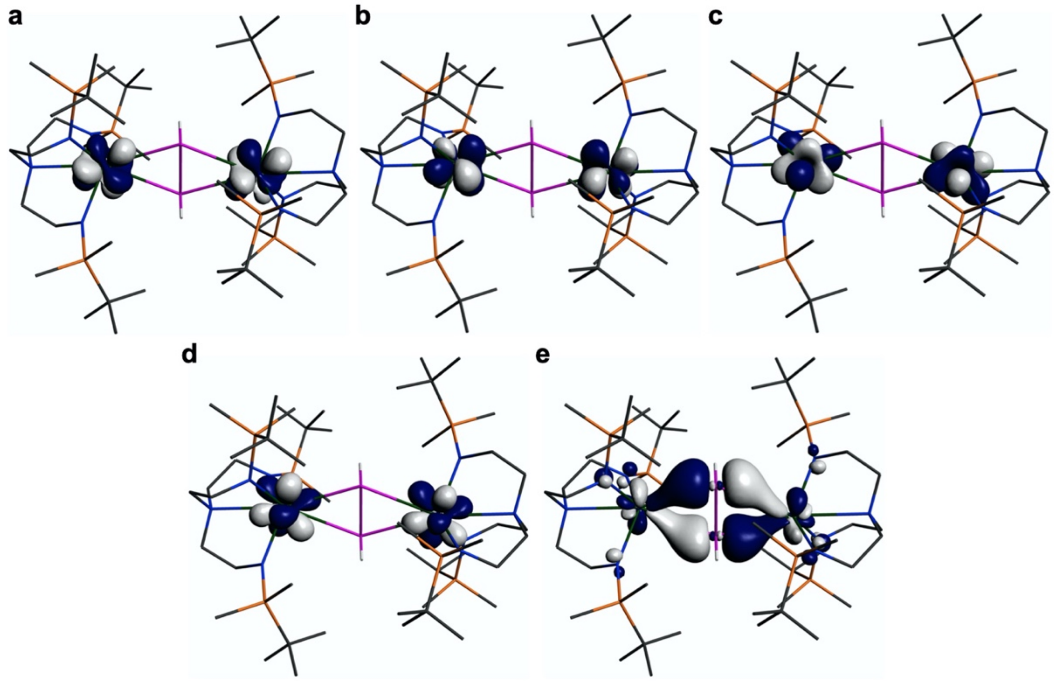

Supplementary Figure 61. Selected molecular orbitals of **8**. a) HOMO (381a, −2.857 eV), b) HOMO−1 (380a, −2.860 eV), c) HOMO−2 (379a, −2.903 eV), d) HOMO−3 (378a, −2.916 eV), e) HOMO−4 (377a, −4.242 eV).

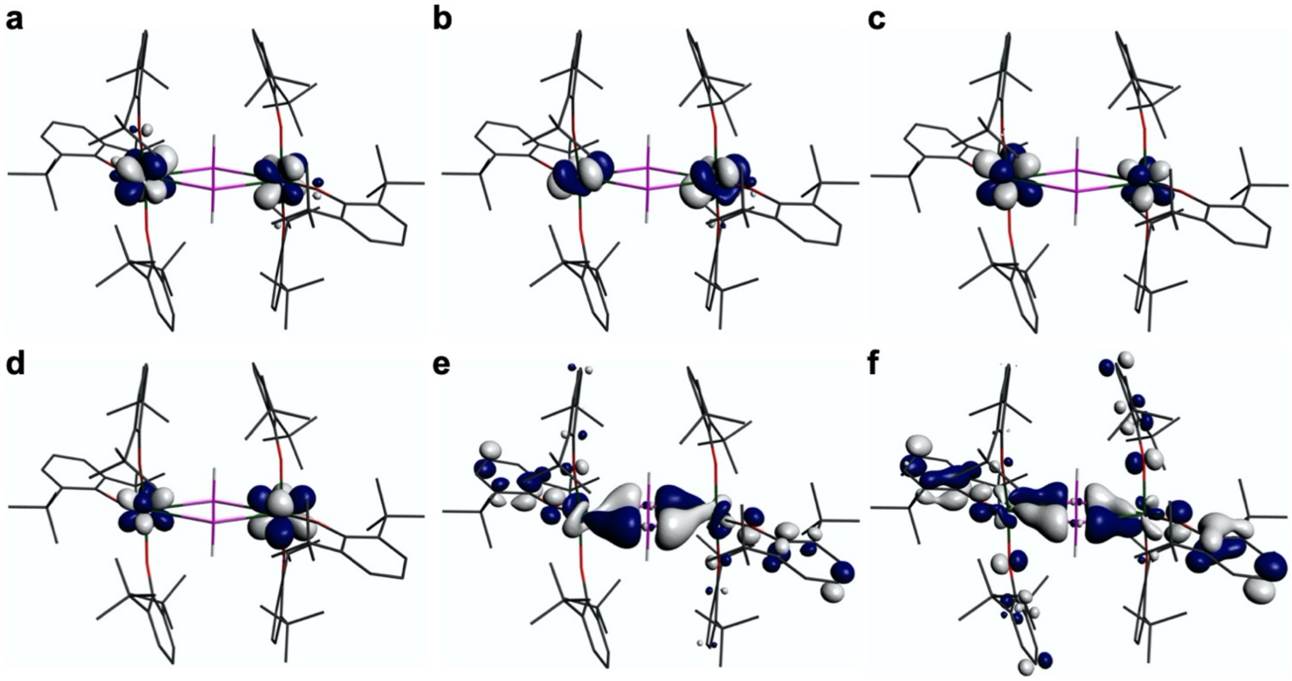

Supplementary Figure 62. Selected molecular orbitals of **9**. a) HOMO (449a, −3.429 eV), b) HOMO−1 (448a, −3.451 eV), c) HOMO−2 (447a, −3.590 eV), d) HOMO−3 (446a, −3.607 eV), e) HOMO−4 (445a, −4.963 eV), f) HOMO−5 (443a, −5.014 eV).

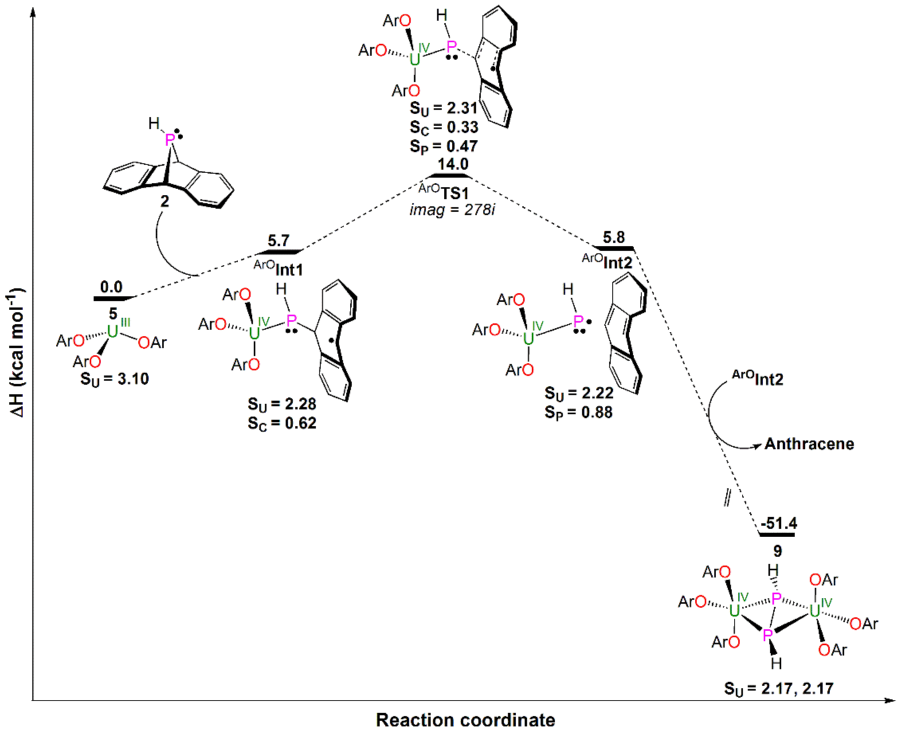

Supplementary Figure 63. Computed enthalpy profile (kcal mol-1) at room temperature for the reaction of **5** with **2** to give **9**. The unpaired electron spin density values are indicated for each uranium compound.

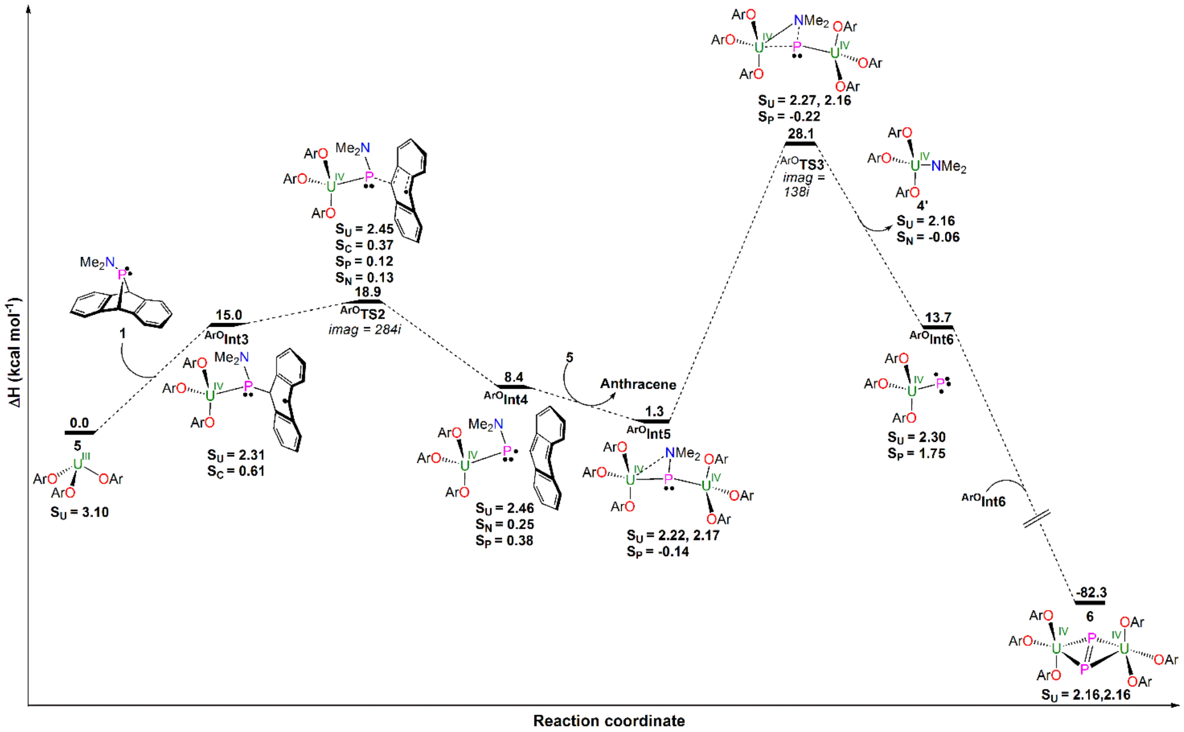

Supplementary Figure 64. Computed enthalpy profile (kcal mol-1) at room temperature for the reaction of **5** with **1** to give **4'** and **6**. The unpaired electron spin density values are indicated for each uranium compound.

SUPPLEMENTARY TABLES

Supplementary Table 1. Computed bond length, indices, charges, and spin density data for 6-9

Cmpd	Bond	Bond lengths and indices		MDC_q charges		MDC_m spin density[b]	
		Dist.	BI[a]	U	P	U	P
**6**	U-P	2.7817	1.03	2.83	−1.17	2.17	0.08
	U-P	2.7817	1.03	2.83	−1.17	2.17	0.08
	U-P	2.8624	0.83				
	U-P	2.8630	0.83				
	P-P	2.0748	1.40				
**7**	U-P	2.8722	1.21	2.92	−2.54	2.25	0.01
	U-P	2.8777	1.22	2.86		2.30	
**8**	U-P	3.0217	0.68	2.89	−1.14	2.26	0.01
	U-P	3.0226	0.68	2.89	−1.13	2.26	0.01
	U-P	3.0553	0.68				
	U-P	3.0577	0.68				
	P-P	2.2354	1.10				
**9**	U-P	2.9150	0.82	2.51	−0.99	2.26	−0.03
	U-P	2.9150	0.82	2.52	−0.99	2.24	−0.03
	U-P	2.9929	0.69				
	U-P	2.9929	0.69				
	P-P	2.2279	1.01				

[a] Nalewajski-Mrozek bond indices. [b] Positive value is accumulation of electron spin density, negative value is a deficit of electron spin density.

Supplementary Table 2. Quantum Theory of Atoms In Molecules data for the U-P and P-P bonds in 6-9

Cmpd	Bond	ρ^a	$\nabla^2\rho^b$	H^c	ε^d
**6**	U-P	0.06	0.08	−0.02	0.19
	U-P	0.06	0.08	−0.02	0.19
	U-P	0.05	0.08	−0.01	0.76
	U-P	0.05	0.08	−0.01	0.76
	P-P	0.13	−0.12	−0.10	0.10
**7**	U-P	0.05	0.07	−0.02	0.20
	U-P	0.05	0.06	−0.02	0.30
**8**	U-P	0.04	0.05	−0.01	0.28
	U-P	0.04	0.05	−0.01	0.27
	U-P	0.04	0.05	−0.01	0.28
	U-P	0.04	0.05	−0.01	0.27
	P-P	0.10	−0.07	−0.07	0.36
**9**	U-P	0.05	0.06	−0.01	0.18
	U-P	0.04	0.06	−0.01	0.57
	U-P	0.05	0.06	−0.01	0.17
	U-P	0.04	0.06	−0.01	0.57
	P-P	0.10	−0.08	−0.07	0.37

[a] Topological electron density. [b] Laplacian. [c] Electronic energy density. [d] Bond ellipticity.

Supplementary Table 3. Spin energetics for Int2

Spin states	ΔH (kcal/mol)	Spin density	
		**U**	**P**
S = 3/2	0.0	2.22	0.92
S = 1/2	7.5	0.11	0.89

Supplementary Table 4. Spin energetics for ^{ArO}Int2

Spin states	ΔH (kcal/mol)	Spin density	
		**U**	**P**
S = 3/2	0.0	2.22	0.88
S = 1/2	12.1	0.77	0.28

Supplementary Table 5. Spin energetics for Int6

Spin states	ΔH (kcal/mol)	Spin density	
		**U**	**P**
S = 2	0.0	2.27	1.81
S = 1	4.7	2.15	-
S = 0	9.3	-	-

Supplementary Table 6. Spin energetics for ^{ArO}Int6

Spin states	ΔH (kcal/mol)	Spin density	
		**U**	**P**
S = 2	0.0	2.30	1.75
S = 1	4.7	2.16	-
S = 0	13.7	-	-
